# Control of Neuronal Excitability by Cell Surface Receptor Density and Phosphoinositide Metabolism

**DOI:** 10.3389/fphar.2021.663840

**Published:** 2021-04-21

**Authors:** Martin Kruse, Rayne J. Whitten

**Affiliations:** ^1^ Department of Biology, Bates College, Lewiston, ME, United States; ^2^ Program in Neuroscience, Bates College, Lewiston, ME, United States

**Keywords:** phosphoinositides, neuronal excitability, superior cervical ganglion, PIP_2_, ion channel

## Abstract

Phosphoinositides are members of a family of minor phospholipids that make up about 1% of all lipids in most cell types. Despite their low abundance they have been found to be essential regulators of neuronal activities such as action potential firing, release and re-uptake of neurotransmitters, and interaction of cytoskeletal proteins with the plasma membrane. Activation of several different neurotransmitter receptors can deplete phosphoinositide levels by more than 90% in seconds, thereby profoundly altering neuronal behavior; however, despite the physiological importance of this mechanism we still lack a profound quantitative understanding of the connection between phosphoinositide metabolism and neuronal activity. Here, we present a model that describes phosphoinositide metabolism and phosphoinositide-dependent action potential firing in sympathetic neurons. The model allows for a simulation of activation of muscarinic acetylcholine receptors and its effects on phosphoinositide levels and their regulation of action potential firing in these neurons. In this paper, we describe the characteristics of the model, its calibration to experimental data, and use the model to analyze how alterations of surface density of muscarinic acetylcholine receptors or altered activity levels of a key enzyme of phosphoinositide metabolism influence action potential firing of sympathetic neurons. In conclusion, the model provides a comprehensive framework describing the connection between muscarinic acetylcholine signaling, phosphoinositide metabolism, and action potential firing in sympathetic neurons which can be used to study the role of these signaling systems in health and disease.

## Introduction

Phosphoinositides are signaling molecules that affect different aspects of neuronal activity, ranging from regulating ion channel activity (Brown and Adams, 1980; Suh and Hille, 2002; Kruse et al., 2012; Hille et al., 2015), controlling neurotransmitter vesicle docking and release (Di Paolo and De Camilli, 2006; Martin, 2014) as well as endocytosis (Cremona and De Camilli, 2001; Posor et al., 2014), to organization of the cytoskeleton (Saarikangas et al., 2010).

One of the most important members of the phosphoinositide family is phosphatidylinositol 4,5-bisphosphate (PI(4,5)P_2_) (Saheki and De Camilli, 2017). PI(4,5)P_2_ is critically involved in the aforementioned processes and its levels can be depleted by more than 90% within seconds through activation of phospholipase C (PLC) in neurons, thereby altering neuronal activity (Reiner and Levitz, 2018; Weis and Kobilka, 2018). Phosphoinositide metabolism is highly dynamic and resynthesis of PI(4,5)P_2_ occurs rapidly after the end of a PLC-activating stimulus via phosphorylation of phosphatidylinositol (PI) and phosphatidylinositol 4-phosphate (PI(4)P) through phosphatidylinositol 4- and 5-kinases, respectively (Willars et al., 1998; Cantley, 2002; Kruse et al., 2016). Interestingly, kinetics of PI(4,5)P_2_ resynthesis after depletion are dependent on the cell type. Studies performed on human embryonic kidney cells have reported durations of 120 s for the restoration of PI(4,5)P_2_ levels after activation of muscarinic acetylcholine receptors type 1 (M_1_R) (Falkenburger et al., 2010a; Dickson et al., 2014; Dickson et al., 2016). In contrast, similar experiments performed on rat superior cervical ganglion (SCG) neurons reported resynthesis of PI(4,5)P_2_ in less than 60 s (Kruse et al., 2016). These differences provide evidence for the hypothesis that different types of cells have adjusted kinetics of PI(4,5)P_2_ synthesis based on their physiological needs.

Work by many different groups has established a strong correlation between electrical activity of neurons and the control of ion channels by PI(4,5)P_2_. For instance, members of the KCNQ family of voltage-gated potassium channels have been shown to be dependent on PI(4,5)P_2_ and to be in control of electrical excitability of different types of neurons including sympathetic neurons such as superior cervical ganglion neurons (Suh and Hille, 2002; Brown et al., 2007; Hernandez et al., 2009; Zaika et al., 2011; Vivas et al., 2014). Members of the transient receptor potential (TRP) channel family as well as potassium channels have been shown to be regulated by breakdown of PI(4,5)P_2_ through phospholipase C in photoreceptors of *Drosophila* (Hardie et al., 2001; Hardie et al., 2004; Krause et al., 2008; Raghu and Hardie, 2009; Hardie and Franze, 2012; Randall et al., 2015; Liu et al., 2018), and lipid transfer proteins involved in phosphoinositide metabolism have been shown to be critical for the functionality of *Drosophila* photoreceptors (Yadav et al., 2015; Cockcroft et al., 2016; Cockcroft and Raghu, 2016; Nath et al., 2020; Basak et al., 2021). In addition, several voltage-gated Ca^2+^ channels are modulated in their activity by PI(4,5)P_2_ levels and influence neuronal excitability as well (Gamper et al., 2004; Suh et al., 2012; Hille et al., 2015). Lastly, while PI(4,5)P_2_ can regulate many ion channels directly, work by Balla and colleagues has provided evidence for a critical role of PI(4,5)P_2_ in control of Schwann cell activity and myelination (Alvarez-Prats et al., 2018; Baba et al., 2020). Many of the biophysical parameters of the interaction between PI(4,5)P_2_ and the aforementioned ion channels have been characterized in detail, however, we still lack an understanding of how dynamic changes of PI(4,5)P_2_ levels influence neuronal excitability. What fraction of total PI(4,5)P_2_ depletion is necessary to evoke action potential firing and how do kinetics of PI(4,5)P_2_ breakdown and resynthesis influence neuronal activity? Currently, these questions can’t be addressed experimentally as we lack tools to monitor phosphoinositide levels in a quantitative manner in a living neuron while also observing its action potential firing at the same time.

One solution for this problem is the development of mathematical models that allow for the simulation of both phosphoinositide metabolism and electrical activity of a neuron. Recently, several different groups have generated mathematical descriptions of phosphoinositide synthesis and breakdown in different types of cells including neurons (Xu et al., 2003; Brown et al., 2008; Dickson et al., 2013; Falkenburger et al., 2013; Hille et al., 2014; Kruse et al., 2016), however, all of these models lack a mathematical description of electrical activity. Recently, Mergenthal et al. published a first report of a mathematical model describing cholinergic modulation of action potential firing in CA1 pyramidal neurons (Mergenthal et al., 2020), however, we still lack such quantitative descriptions of phosphoinositide metabolism and its role for action potential firing for other types of neurons. Here, we report the generation of a new mathematical model that provides a description of phosphoinositide metabolism and muscarinic acetylcholine receptor signaling as well as action potential firing and underlying ion channel activity in rat superior cervical ganglion neurons. The model takes phosphoinositide dependence of ion channels into account and allows for a quantitative exploration of the connection between lipid metabolism, G protein-coupled receptor signaling, and action potential firing in a sympathetic neuron.

## Materials and Methods

We used a model of phosphoinositide metabolism of rat superior cervical ganglion neurons previously published by our group as well as a model of action potential firing in these neurons published by Zaika et al. as a starting point to develop a new model that describes neuronal activity of sympathetic neurons as phosphoinositide-dependent action potential firing in response to activation of muscarinic acetylcholine receptors (Zaika et al., 2006; Kruse et al., 2016). The following sections describe the procedures performed to acquire needed experimental data for the development of the model.

### Animal Handling

Male adult Sprague-Dawley rats were obtained from The Jackson Laboratory (Bar Harbor, ME). All work with these animals described in this study has been approved and overseen by the Bates College Institutional Animal Care and Use Committee.

### Cell Culture

Human embryonic kidney cells (HEK293, ATCC Cat# CRL-1573, RRID:CVCL_0045) were maintained at 50–70% confluency in Eagle’s Minimum Essential Medium (EMEM, ATCC Cat# 30–2003)) supplemented with 10% (v/v) fetal bovine serum (Life Technologies Cat# 16000044), 100 units/ml Penicillin, and 100 μg/ml Streptomycin (Life Technologies Cat# 15140148) at 37 C and 5% CO_2_. Transfection of HEK293 cells was carried out with Lipofectamine 3000® according to manufacturer instructions (Life Technologies Cat#L3000001).

### Plasmids

A pcDNA3-PH_PLCδ1_-RFP plasmid was provided by Kees Jalink (Netherlands Cancer Institute, Amsterdam, Netherlands) while Dr-VSP-IRES-GFP (encoding for Dr-VSP cloned into the pIRES2-GFP vector) was provided by Yasushi Okamura (Osaka University, Osaka, Japan). Plasmids encoding for rat KCNQ2/3 channels cloned into the pcDNA3 expression vector were obtained from D. McKinnon (State University of New York, Stony Brook, NY), M_1_R-YFP (coding sequence for mouse muscarinic acetylcholine receptor type one fused N-terminally to the coding sequence for the yellow fluorescent protein using the vector pEYFPN1) was provided by N. Nathanson (University of Washington, Seattle, WA), and a pcDNA3-M_1_R encoding plasmid was obtained from the cDNA Resource Center (Bloomsberg, PA). A plasmid encoding for the human KCNMA1 (Maxi-K α) subunit expressed via the pcDNA3 vector backbone was provided by R. Aldrich (University of Texas, Austin, TX). All expression plasmids utilized a CMV promotor for expression of cDNAs.

### Solution and Reagents

Cells were superfused throughout all electrophysiological and confocal imaging experiments with extracellular recording solution (150.0 mM NaCl, 2.5 mM KCl, 2.0 mM CaCl_2_, 1.0 mM MgCl_2_, 10.0 mM HEPES, and 8.0 mM glucose, adjusted to pH 7.4 with NaOH). Pipette solution for electrophysiological recordings of KCNQ2/3 channels contained 175.0 mM KCl, 5.0 mM MgCl_2_, 5.0 mM HEPES, 0.1 mM K_4_BAPTA, 3.0 mM Na_2_ATP, and 0.1 mM Na_3_GTP, adjusted to pH 7.2 with KOH. Pipette solution for patch clamp recordings of KCNMA1 channels contained 95.0 mM KCl, 20.0 mM K_4_-BAPTA, 19.8 mM CaCl_2_, 1.0 mM MgCl_2_, 5.0 mM HEPES, 3.0 mM Na_2_ATP, and 0.1 mM Na_3_GTP, adjusted to pH 7.2 with KOH. All chemicals used in this study were obtained from Sigma-Aldrich (St. Louis, MO) except for TRIzol reagent (Life Technologies Cat# 15596018) and Fluo-4 AM (Life Technologies Cat# F14201).

### Electrophysiology

Whole-cell patch clamp recordings were carried out using an EPC10 patch clamp amplifier (HEKA Elektronik, EPC 10 USB Patch Clamp Amplifier, RRID:SCR_018399) and Patchmaster software for data acquisition (HEKA Elektronik, software version 2 × 90.5). Recording pipettes were manufactured from borosilicate glass (Sutter Instruments Cat# BF150-110-10) and fire-polished to a final resistance of 2–3 MΩ using a Sutter P-97 pipetter puller (Sutter P-97/PC Pipette Puller, RRID:SCR_018636). All recordings were performed at room temperature and under constant superfusion of cells with extracellular recording solution (see Solutions and Reagents) at a flow rate of 2 ml/min. Currents were sampled at 5 kHz, and series resistance was compensated by 50–70% after compensation of fast and slow capacitances. Low- and high-pass filters were set to 2.8 and 10 kHz, respectively. KCNQ- or KCNMA1-channels were activated by a 500 ms long depolarization to a membrane potential of -20 mV from a holding potential of -60 mV. Channels were activated every 2 s. Dr-VSP was activated from a holding potential of -60 mV via three 400 ms long depolarizations to a membrane potential of 100 mV with a return to the holding potential of -60 mV for 100 ms between each depolarization. Recordings were analyzed using IGOR Pro 7.0 (WaveMetrics Inc., IGOR Pro, RRID:SCR_000325) and Patcher’s Power Tools 2.18 (Patchers Power Tools, RRID:SCR_001950).

### Confocal Imaging

All imaging experiments were carried out using a Leica SP8 microscope (Leica Microsystems Inc., Leica SP8 LIGHTNING confocal microscope, RRID:SCR_018169) at room temperature. Data acquisition was performed using LAS 4.1 software (Leica Microsystems Inc.). Cells were constantly superfused with extracellular recording solution (see Solutions and Reagents) at a flow rate of 2 ml/min. For calcium imaging experiments, cells were loaded with Fluo-4 AM (Life Technologies Cat# F14201) in extracellular recording solution prior to experiments according to manufacturer instructions. Fluo-4 was excited using light with a wavelength of 494 nm generated by a STELLARIS white light laser (Leica Microsystems Inc.) and emission was monitored between 510 and 550 nm by a hybrid detector (Leica Microsystems Inc.) while RFP was excited using light of a wavelength of 558 nm and its emission was detected between 580 and 600 nm. All data was acquired using a 63× oil-immersion objective (Leica Microsystems Inc.). Image analysis was performed with Fiji software 2.1.0 (https://imagej.net/Fiji).

### Molecular Biology

Total RNA was isolated from superior cervical ganglia, heart, brain, and colon of adult male rats with TRIzol reagent (Life Technologies Cat# 15596018) according to manufacturer instructions. Total RNA underwent reverse transcription using the ProtoScript II First Strand cDNA Synthesis Kit (New England Biolabs Cat# E6560S) with D(T)_23_ VN oligonucleotides based on manufacturer instructions. Subsequently, synthesized cDNA was used in polymerase chain reactions with Q5 High-Fidelity DNA polymerase (New England Biolabs Cat# M0491S) and oligonucleotides for α- and β- subunits of rat KCNMA1 channels. Oligonucleotide sequences were as follows: rat KCNMA1 subunit (fwd: 5-CGA​GAC​GGC​TCT​TAG​AAT​GAG​CAG​C-3, rev: 5-CAT​TGG​CTG​CAA​TAA​ACC​GCA​AGC​C-3), rat KCNMB1 subunit (fwd: 5-ATG​GGG​AAG​AAG​CTG​GTG​ATG-3, rev: 5-CTG​GTA​CAC​AAC​ACT​GGT​CTC-3), rat KCNMB2 subunit (fwd: 5-CCG​GAC​CTC​TTC​ATC​TTA​CAG-3, rev: 5-TCT​TCA​TGG​TCT​CTT​CTG​TGT​G-3), rat KCNMB3 subunit (fwd: 5-GAA​TCA​AAC​TGC​ACC​ACT​GTC-3, rev: 5-CCT​AAC​CAA​ACC​AAC​AAT​CAG​AG-3), and rat KCNMB4 subunit (fwd: 5-ATG​GCG​AAG​CTC​AGG​GTG​TCT​TAC​G-3, rev: 5-GAG​GAC​CAC​GAT​GAG​GAC​ACC​C-3).

### Model Development

The model was developed in R and all programming code is publicly available (https://github.com/Martin-Kruse/SCG-PI-excitability). The development and evaluation of the individual aspects of the model is described in the Results section.

## Results

### Expression of KCNMA1 and KCNMB Subunits in Rat Superior Cervical Ganglia

**FIGURE 1:**
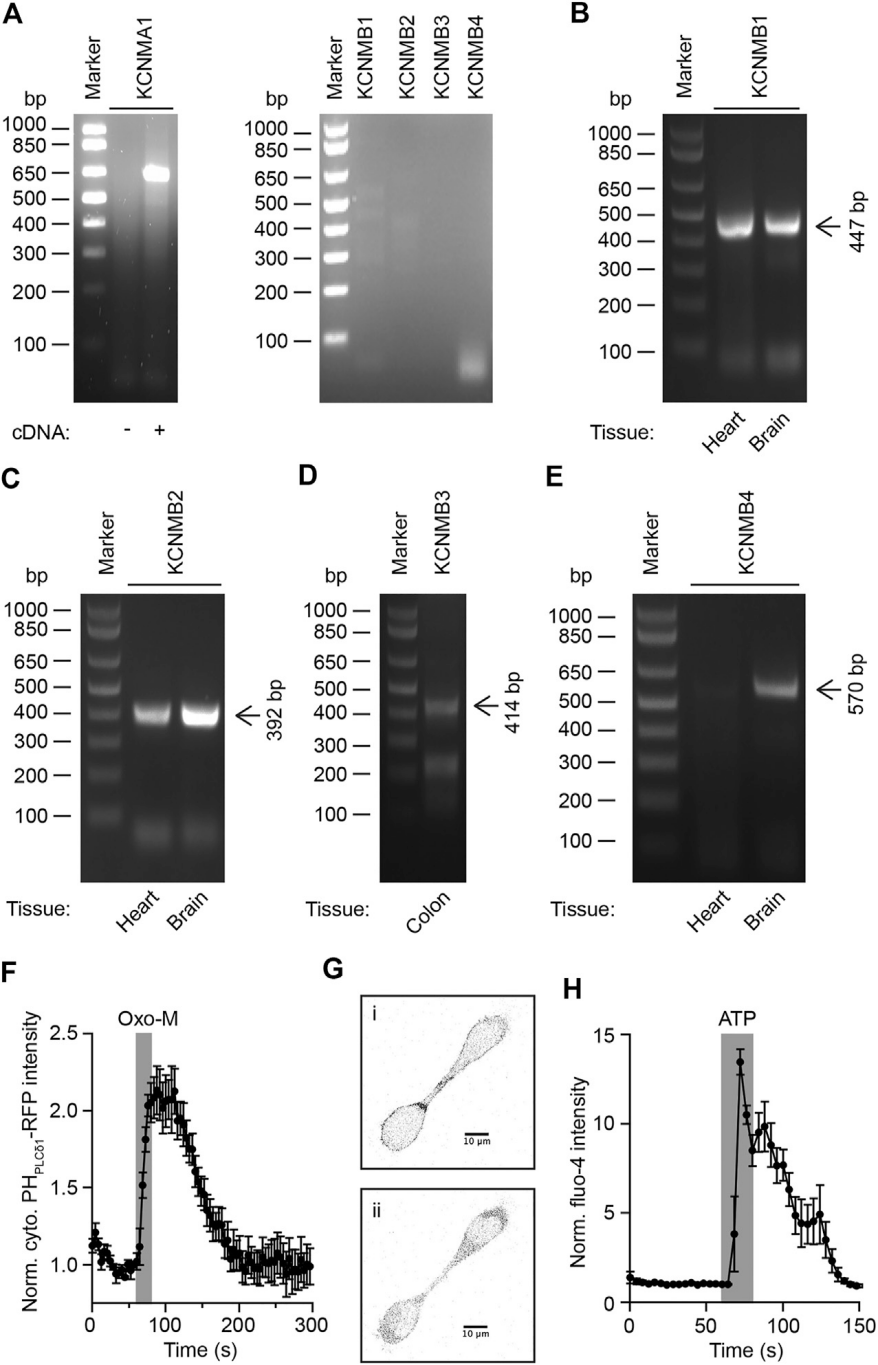
Expression analysis of KCNMA1 channel complexes in rat superior cervical ganglion neurons. (**A)** **Left:** RT-PCR on cDNA generated from total RNA isolated from rat SCG with oligonucleotides for KCNMA1 channel α-subunit. **Right:** Same as on left, but with oligonucleotides for KCNMA1 channel β-subunits. **(B)** - **(E)**. RT-PCR on cDNA generated from total RNA isolated from indicated rat tissues with oligonucleotides for indicated KCNMB subunits. Arrows indicate expected molecular weight for PCR fragments. **(F)**. Normalized cytoplasmic RFP-intensity in HEK293 cells expressing PH_PLCδ1_-RFP and mouse M_1_R in response to stimulation of cells with 10 μM Oxo-M (*n* = 5). Error bars indicate SEM. Traces were normalized to the last timepoint before application of Oxo-M. **(G)** . Representative gray-scale images of HEK293 cells expressing PH_PLCδ1_-RFP before 1) and after 2) activation of muscarinic acetylcholine receptors by 10 μM Oxo-M. **(H)**. Normalized fluo-4 intensity in HEK293 cells in response to stimulation of cells with 100 μM ATP (*n* = 6). Error bars indicate SEM. Traces were normalized to the last timepoint before application of ATP.

Previous electrophysiological recordings of rat superior cervical ganglion neurons showed a critical role for both KCNQ and KCNMA1 potassium channels in controlling excitability of SCG neurons (Brown et al., 1982; Suh and Hille, 2002; Vivas et al., 2014). Both of these channel families have been shown to be regulated by PI(4,5)P_2_, but while this dependence relies on an interaction of PI(4,5)P_2_ with the α-subunits of KCNQ channels, any potential regulation of KCNMA1 channels by phosphoinositides seems to be caused by an interaction with KCNMA1 β-subunits (Vaithianathan et al., 2008; Hille et al., 2015). In order to develop a mathematical description of electrical activity and its regulation by phosphoinositides in SCGs, we needed to determine if any KCNMA1 channel β-subunits are expressed in SCG of adult rats. For this purpose, we isolated total RNA from SCGs and reverse transcribed it into cDNA. Polymerase chain reactions with oligonucleotides for rat KCNMA1 channel α-subunit showed a band at ∼ 650 bp, consistent with the predicted fragment size of 641 bp (Figure 1A, left panel). This result indicates expression of KCNMA1 channel α-subunits in our preparation and agrees with previous studies (Vivas et al., 2014; Vivas et al., 2017). Polymerase chain reactions on cDNA from rat SCGs with oligonucleotides for KCNMA1 channel β-subunits showed no detectable fragments, indicating an absence of KCNMA1 channel β subunits in rat superior cervical ganglia neurons (Figure 1A, right panel). Given the absence of detectable PCR products for all four KCNMB subunits in cDNA isolated from total RNA of SCGs, we decided to isolate total RNA from rat tissues that had previously been described to express KCNMB subunits either in rodents or humans (Chang et al., 1997; Xia et al., 1999; Bautista et al., 2009; Fagerberg et al., 2014; Whitmire et al., 2017). Reverse transcription of these RNAs into cDNA with subsequent PCR on the cDNA showed PCR products of the expected size of 447 bp for KCNB1 in heart and brain (Figure 1B), a fragment of an expected size of 392 bp for KCNMB2 in heart and brain (Figure 1C), a fragment of an expected size of 414 bp for KCNMB3 in colon (Figure 1D), and a fragment of an expected size of 570 bp for KCNMB4 in brain (Figure 1E). We concluded that the chosen oligonucleotides for the detection of cDNA for the four KCNMB subunits are capable of allowing for amplification of PCR products for all four KCNMB subunits, thereby supporting our interpretation that KCNMB subunits are not expressed in SCGs.

### Analysis of PI(4,5)P_2_-Dependence of rat KCNMA1 Channels

We now turned our attention to the question of whether rat KCNMA1 channels depend on PI(4,5)P_2_ for their activity. To verify that we can successfully deplete plasma membrane PI(4,5)P_2_ in a transient manner, we transfected HEK293 cells with expression plasmids for mouse muscarinic acetylcholine receptor type I and PH_PLCδ1_-RFP. Application of 10 µM oxotremorine methiodide (Oxo-M) for 20 s caused strong translocation of PH_PLCδ1_-RFP from the plasma membrane into the cytoplasm, indicating net depletion of PI(4,5)P_2_ at the plasma membrane (Figures 1F,G). To verify our result of successful activation of phospholipase C with a second, independent method, we loaded HEK293 cells with the membrane-permeable calcium-sensitive fluorescent dye Fluo-4-AM and activated endogenous purinergic receptors by a 20 s application of 100 µM ATP while monitoring Fluo-4-indicated cytoplasmic calcium levels. We observed a strong increase in fluorescence intensity upon application of ATP, indicating an activation of phospholipase C (PLC) and depletion of PI(4,5)P_2_ at the plasma membrane while generating cytoplasmic inositol 1,4,5-trisphosphate and triggering calcium release from the endoplasmic reticulum (Figure 1H).

**FIGURE 2:**
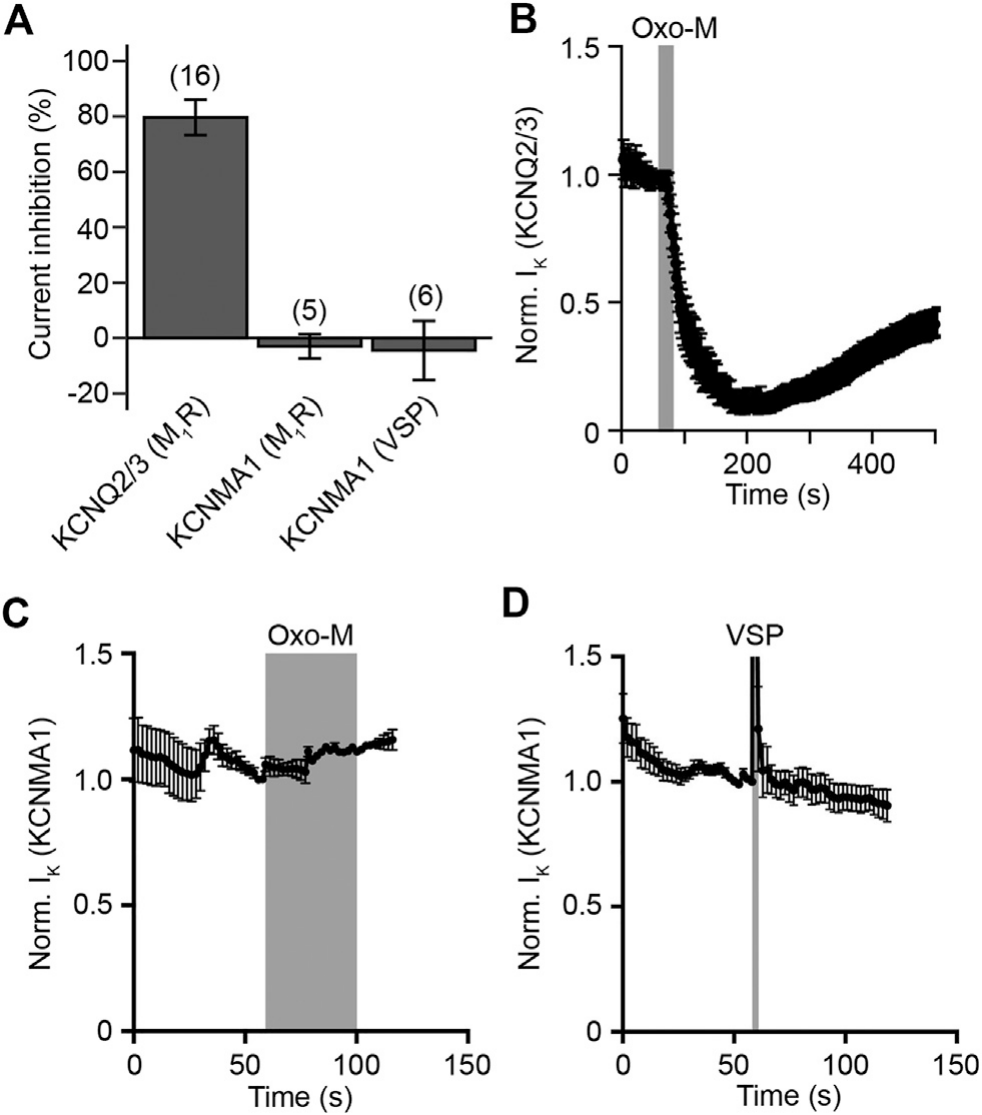
Electrophysiological characterization of PI(4,5)P2-dependence of KCNMA1 channel α-subunits. **(A)**. Current inhibition of KCNQ2/3 or KCNMA1 channels in response to activation of M_1_R or Dr-VSP. Numbers in brackets indicate number of experiments. Error bars indicate SEM. **(B)**. Normalized KCNQ2/3-mediated current in HEK293 cells in response to stimulation of expressed mouse muscarinic acetylcholine receptors type 1 for 20 s with 10 μM Oxo-M (*n* = 16, error bars indicate SEM). Traces were normalized to the last timepoint before application of Oxo-M. **(C)**. Normalized KCNMA1 channel α-subunit-mediated current in HEK293 cells in response to stimulation of expressed muscarinic acetylcholine receptors type 1 for 40 s with 10 μM Oxo-M (*n* = 5, error bars indicate SEM). Traces were normalized to the last timepoint before application of Oxo-M. **(D)**. Normalized current recorded from HEK293 cells transiently transfected with Dr-VSP and KCNMA1 channel α-subunits (*n* = 6, error bars indicate SEM). Grey bar indicates time of depolarization to 100 mV to activate Dr-VSP. Traces were normalized to the last timepoint before activation of Dr-VSP.

Having shown that SCG neurons seem to express only KCNMA1 channel α-, but not β-subunits, and that activation of PLC in HEK293 cells causes net depletion of PI(4,5)P_2_, we asked whether a reduction of PI(4,5)P_2_ levels would cause an alteration of KCNMA1 channel activity. We first expressed KCNQ2/3 channels and M_1_R in HEK293 cells and measured KCNQ2/3-mediated potassium current amplitudes by whole cell patch-clamp recordings. These channels have been shown to be strongly dependent on PI(4,5)P_2_ and can therefore serve as a positive control for electrophysiological detection of net PI(4,5)P_2_ depletion (Suh and Hille, 2002; Brown et al., 2007; Kruse et al., 2012; Dickson et al., 2013). As expected, activation of M_1_R reduced KCNQ2/3-mediated current amplitudes by 79.7 ± 6.4% (mean ± SEM, *n* = 16, *p* < 0.001, one-sample *t*-test), a finding that indicates strong net depletion of PI(4,5)P_2_ by M_1_R activation in HEK293 cells (Figures 2A,B). Next, we transfected HEK293 cells with expression plasmids for M_1_R and KCNMA1 channels. Measurement of KCNMA1 channel mediated current amplitudes before and after activation of M_1_R by 10 µM Oxo-M showed no significant change in current amplitude (-3.0 ± 4.4%, mean ± SEM, *n* = 5, *p* = 0.231, one-sample *t*-test) (Figures 2A,C). While this result indicated that KCNMA1 channels do not require PI(4,5)P_2_ for full activity at the plasma membrane, we decided to repeat this test by using a different method of net PI(4,5)P_2_ depletion. We transfected HEK293 cells with a voltage-sensing lipid phosphatase, Dr-VSP, which has been shown to cause strong net depletion of PI(4,5)P_2_ upon a depolarizing voltage pulse (Hossain et al., 2008; Iwasaki et al., 2008; Halaszovich et al., 2009; Falkenburger et al., 2010b). Interestingly, activation of Dr-VSP showed again no significant alteration of KCNMA1 channel activity (-4.5 ± 10.7%, mean ± SEM, *n* = 6, *p* = 0.328, one-sample *t*-test) (Figures 2A,D). We concluded that net depletion of PI(4,5)P_2_ does not seem to significantly alter KCNMA1 channel activity.

### Mathematical Description of Ion Channel Conductances in Rat SCG Neurons

In order to develop a mathematical model of action potential firing in superior cervical ganglion neurons, we expanded a previously published model by Zaika et al. (2006). Similar to their model, we included a voltage-gated sodium conductance (Traub et al., 1991), a delayed rectifier potassium (KDR) conductance (Migliore et al., 1995), a high-threshold voltage-gated L-type calcium channel conductance (Migliore et al., 1995), a calcium-dependent KCNMA1 channel conductance (Moczydlowski and Latorre, 1983; Migliore et al., 1995), and a M-current conductance (Borg-Graham, 1991; Migliore et al., 1995). In addition to these conductances, we also included a low-threshold L-type calcium conductance (Somjen et al., 2008) and mathematical descriptions of a sodium-calcium exchanger as well as a Ca^2+^ ATPase to simulate intracellular Ca^2+^ clearance mechanisms (Quadroni and Knopfel, 1994). Changes of the membrane potential were calculated by the following equation, where, *I*_*KM*_, *I*_*BK*_, *I*_*KDR*_, *I*_*Na*_, *I*_*Ca(low)*_, *I*_*Ca(high)*_, *I*_*CaATPase*_, *I*_*NaCaEx*_, and *I*_*L*_ are specific ionic currents representing KCNQ-, KCNMA1-, KDR-, high-threshold Ca^2+^-, low-threshold Ca^2+^-channels, respectively, as well as Ca^2+^-ATPase -, Na^+^-Ca^2+^-exchanger activities, and leak conductances. All parameters and constants used in the following equations are listed in Supplemental Table S1.

$$\begin{array}{c} C\frac{dV}{dt}=\;-I_{KM}-I_{BK}-I_{KDR}-I_{Na}-I_{Ca\left(\mathrm{high}\right)}-I_{Ca\left(\mathrm{low}\right)}-I_{\mathrm{CaATPase}}-I_{\mathrm{NaCaEx}}-I_{L} \end{array}$$ (1)

In this equation, V represents the membrane potential, t is time, and C is the membrane capacitance. The ionic currents are described by the following equations:

$$\begin{array}{c} I_{KM}=g_{KM}k\;\left(V-E_{K}\right) \end{array}$$ (2)

$$\begin{array}{c} I_{BK}=g_{BK}o\;\left(V-E_{K}\right) \end{array}$$ (3)

$$\begin{array}{c} I_{KDR}=g_{KDR}\;n^{3}\;l\;\left(V-E_{K}\right) \end{array}$$ (4)

$$\begin{array}{c} I_{Na}=g_{Na}\;m^{3}\;h\;\left(V-E_{Na}\right) \end{array}$$ (5)

$$\begin{array}{c} I_{Ca\left(high\right)}=g_{calbar}\;c^{2}\;\left(k_{i}\frac{k_{i}}{k_{i}+\frac{Ca_{c}}{1000}}\right)\cdot \left(-f\;\left(1-\left(\frac{\left(\frac{Ca_{c}}{1000}\right)}{Ca_{ex}}\right)\cdot \exp\frac{V}{f}\;\right)\cdot efun\left(\frac{V}{f}\right)\right) \end{array}$$ (6)

$$\begin{array}{c} I_{Ca\left(low\right)}=g_{calbar2}\;c2\;\left(k_{i}\frac{k_{i}}{k_{i}+\frac{Ca_{c}}{1000}}\right)\cdot \left(-f\;\left(1-\left(\frac{\left(\frac{Ca_{c}}{1000}\right)}{Ca_{ex}}\right)\cdot \exp\frac{V}{f}\;\right)\cdot efun\left(\frac{V}{f}\right)\right) \end{array}$$ (7)

$$\begin{array}{c} I_{CaATPase}=K2f_{ATPase}\left(\frac{f_{ATPase}\cdot \frac{Ca_{c}}{1000}}{f_{ATPase}\cdot \frac{Ca_{c}}{1000}+b_{ATPase}}\right) \end{array}$$ (8)

$$\begin{array}{c} I_{NaCaEx}=\;-K2f_{ex}\cdot \left(Na_{C}^{3}\cdot Ca_{ex}\cdot \exp\left(E_{1}\cdot V\right)-Na_{ex}^{3}\cdot \frac{Ca_{c}}{1000}\cdot \exp\left(-E_{2}\cdot V\right)\right) \end{array}$$ (9)

$$\begin{array}{c} I_{L}=g_{L}\left(V-E_{L}\right) \end{array}$$ (10)

In these equations, *g*_*x*_ indicates the conductances per unit area of ion channel *x*, and *Ca*_*c*_, *Ca*_*ex*_, *Na*_*C*_, and *Na*_*ex*_ represent the intra- and extracellular concentration of Ca^2+^ and Na^+^, respectively. All activation and inactivation gating variables (described as *x* in the following equation) are expressed as stated in Eq. (11) based on the formulations by Hodgkin and Huxley (1952):

$$\begin{array}{c} \frac{dx}{dt}=\frac{x_{\infty }-x}{\tau_{x}}\; \end{array}$$ (11)

The function “efun” referenced in Eqs. (6, 7) is available in the program code listed under https://github.com/Martin-Kruse/SCG-PI-excitability and is listed in the Supplemental Information. All equations for the description of ion channel activities are listed in the Supplemental Information.

### Mathematical Description of Phosphoinositide Metabolism in Rat SCG Neurons

The mathematical model of phosphoinositide metabolism of rat superior cervical ganglion neurons used in this model has been developed by our research group and has been described in a previous publication (Kruse et al., 2016). We have not modified this model for this study, so we refer the reader to our previous publication for a detailed description of the characteristics and development of this part of the model presented in this publication. Here, we will discuss the most essential principles of this model and introduce the equations of the model that are of greatest relevance for this study. The most relevant parameters for the description of phosphoinositide metabolism in this model are listed in Supplemental Table S2. We refer the reader to our previous publication for a full description of all parameters (Kruse et al., 2016).

The relative contributions of phosphatidylinositol (PI), phosphatidylinositol 4-phosphate (PIP), and phosphatidylinositol 4,5-bisphosphate (PI(4,5)P_2_) to the overall phosphoinositide pool of rat SCG neurons were determined by lipid mass spectrometry (Kruse et al., 2016; Traynor-Kaplan et al., 2017). The kinetics of breakdown of PIP and PIP_2_ by phospholipase C (PLC) after activation of muscarinic acetylcholine receptors type I as well as their resynthesis were measured using fluorescent lipid-binding domains such as P4M for PIP (Hammond et al., 2014), and PH_PLCδ1_ and Tubby-R332H for PI(4,5)P_2_, respectively (van der Wal et al., 2001; Hughes et al., 2007). Changes in levels of active PLC were described by the following equation in the model:

$$\begin{array}{c} \frac{d_{PLC\left(G\alpha GTP\right)}}{dt}=\left(\left(Kf_{PLC_{assoc}}PLC\cdot G\alpha GTP\right)-\left(Kr_{PLC_{assoc}}\cdot PLC\left(G\alpha GTP\right)\right)\right)\\ +\left(Kf_{NE_{G\alpha P}}\cdot G\alpha GDP_{PLC}\right)-\left(Kf_{GTPase_{G\alpha P}}\cdot PLC\left(G\alpha GTP\right)\right) \end{array}$$ (12)

Rates of changes of PIP were calculated using the following equation:

$$\begin{array}{c} \frac{d_{PIP}}{dt}=\left(k4_{rest}\cdot foldPIP_{2}*PI\right)-\left(k4P\cdot PIP\right)-\left(k5k_{rest}\cdot foldPIP_{2}\cdot PIP\right)\\ +\left(k5P_{rest}\cdot foldPIP_{2}\cdot PIP_{2}\right)-\left(PIP\cdot PLC_{efficiency}PIP\right)\cdot \left(PLC_{basal}+K_{PLC}\cdot PLC\left(G\alpha GTP\right)\right)\\ -\left(\left(speed_{P4M_{PIP}}\cdot P4M\cdot PIP\right)-\left(speed_{P4M_{PIP}}\cdot KD_{P4M_{PIP}}\cdot P4M_{PIP}\;\right)\right)\; \end{array}$$ (13)

Similarly, rates of changes of PI(4,5)P_2_ were calculated by the following equation:

$$\begin{array}{c} \frac{d_{PIP_{2}}}{dt}=\left(k5k_{rest}\cdot foldPIP_{2}\cdot PIP\right)-\left(k5P_{rest}\cdot foldPIP_{2}\cdot PIP_{2}\right)-\frac{d_{boundPIP2}}{dt}\\ -\left(\left(speed_{Tubby_{PIP2}}\cdot Tubby\cdot PIP_{2}\right)-\left(speed_{Tubby_{PIP2}}\cdot KD_{Tubby_{PIP2}}\cdot Tubby_{PIP2}\right)\right)\\ -\left(\left(speed_{PH_{PIP2}}\cdot PH_{PLC\delta }\cdot PIP_{2}\right)-\left(KD_{PH_{PIP2}}\cdot speed_{PH_{PIP2}}\cdot PH_{PIP2}\right)\right)\\ -\left(PIP_{2}\cdot (PLC_{basal}+foldPIP_{2}\cdot PLC\left(G\alpha GTP\right)\right))\\ -\left(\left(speed_{PIP2_{KCNQ}}\cdot KCNQ\cdot PIP_{2}\right)-\left(speed_{PIP2_{KCNQ}}\cdot KD_{KCNQ_{PIP2}}\cdot KCNQ_{PIP2}\right)\right)\; \end{array}$$ (14)

The rates of changes of bound PI(4,5)P_2_ were calculated using the following equation:

$$\begin{array}{c} \frac{d_{boundPIP2}}{dt}=\left(\left(\left(-1.0+foldPIP_{2}\right)\cdot speed_{buffering}\right)\cdot PIP_{2}\right)\\ -\left(speed_{buffering}\cdot boundPIP_{2}\right) \end{array}$$ (15)

Rates of changed of free KCNQ channels or KCNQ channels in a complex with PI(4,5)P_2_ were calculated by the following two equations:

$$\begin{array}{c} \frac{d_{KCNQ}}{dt}=\;-\left(\begin{array}{c} \left(speed_{PIP2_{KCNQ}}\cdot KCNQ\cdot PIP_{2}\right)\\ -\left(speed_{PIP2_{KCNQ}}\cdot KD_{KCNQ_{PIP2}}\cdot KCNQ_{PIP2}\right) \end{array}\right) \end{array}$$ (16)

$$\begin{array}{c} \frac{d_{KCNQ_{PIP2}}}{dt}=\left(\begin{array}{c} \left(speed_{PIP2_{KCNQ}}\cdot KCNQ\cdot PIP_{2}\right)\\ -\left(speed_{PIP2_{KCNQ}}\cdot KD_{KCNQ_{PIP2}}\cdot KCNQ_{PIP2}\right) \end{array}\right) \end{array}$$ (17)

### Model Evaluation

In order to generate a model of action potential firing of rat SCG neurons that simulates action potential firing in response to activation of muscarinic acetylcholine receptors, we combined the mathematical descriptions of the aforementioned conductances with our previously published model of phosphoinositide metabolism and muscarinic acetylcholine receptor activity in superior cervical ganglion neurons (Kruse et al., 2016), and added a dependence of KCNQ channels on PI(4,5)P_2_ levels based on previously described affinities of these channels for PI(4,5)P_2_ (Falkenburger et al., 2010b).

**FIGURE 3:**
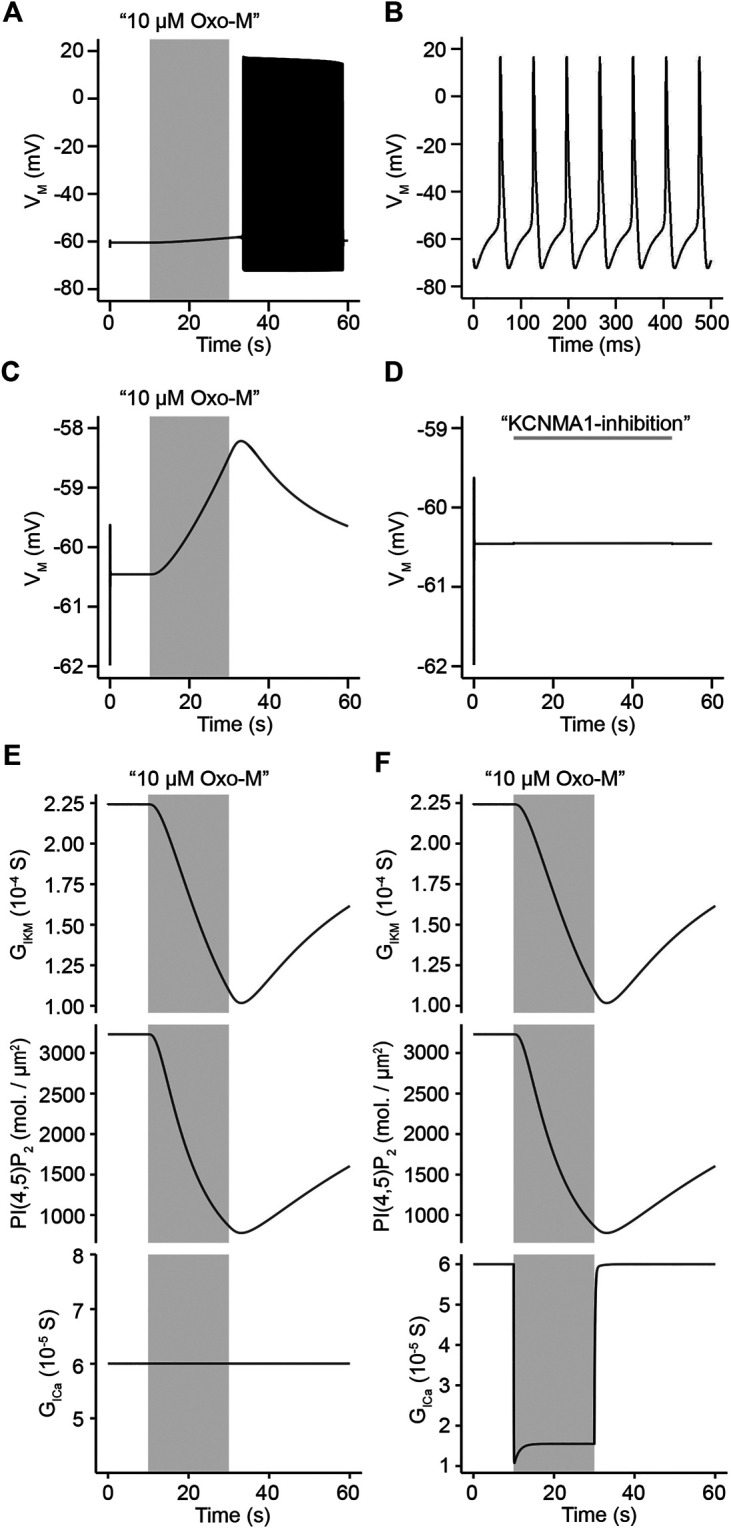
Evaluation of model of action potential firing in rat superior cervical ganglion neurons. **(A)**. Simulated membrane potential of rat SCG neurons in response to stimulation with 10 μM Oxo-M for 20 s. **(B)**. Simulated action potentials representing time period from 40.0 s to 40.5 s in simulation shown in A. **(C)**. Simulation of change in membrane potential to a 20 s application of 10 μM Oxo-M **(top panel)** under a condition of maximum L-type channel conductance over the entire simulation period. **(D)**. Simulated membrane potential of rat SCG neurons over 60 s in response to 100% inhibition of KCNMA1 channel activity from 10 s to 50 s of the simulation. **(E)**. KCNQ2/3 channel conductance **(top panel)**, and PI(4,5)P_2_ density **(middle panel)** under a condition of maximum L-type channel conductance over the entire simulation period **(bottom panel)**. **(F)**. Same as in E, but allowing for modification of L-type channel conductance in response to stimulation with Oxo-M **(bottom panel)**.

The newly generated model reproduced several key properties of electrical activity in SCG neurons. First, the model predicted a stable resting membrane potential of ∼ -60.5 mV and an absence of spontaneous action potential firing without activation of muscarinic acetylcholine receptors (Figure 3A). These properties are in line with experimental observations from rat SCG neurons obtained by perforated patch-clamp recordings from isolated SCG neurons (Vivas et al., 2014). Second, simulated activation of M_1_R causes an initial slow depolarization that eventually causes a train of action potentials lasting for approximately 20–30 s before the membrane potential returns to its resting value (Figure 3A), which again aligns well with previously obtained experimentally observed electrical behavior of rat SCG neurons (Vivas et al., 2014). Lastly, properties of individual simulated action potentials such as threshold of depolarization, firing frequency, and kinetics of de- and repolarization closely resembled action potentials recorded from primary rat SCG neurons (Figure 3B) (Vivas et al., 2014).

Generation of action potential firing in rat SCG neurons has been shown to require a simultaneous inhibition of both KCNQ and KCNMA1 channels (Vivas et al., 2014). Inhibition of KCNQ channels is caused by net depletion of PI(4,5)P_2_ while inhibition of KCNMA1 channels is dependent on a reduction of calcium influx through L-type calcium channels (Vivas et al., 2014; Vivas et al., 2017). We tested our model to determine if it would reproduce these key characteristics of action potential firing in SCG neurons. In order to evaluate this, we first simulated activation of acetylcholine receptors by a 20 s application of 10 μM Oxo-M while clamping the conductance of L-type calcium channels at its starting value, thereby not allowing for a virtual suppression of calcium channel activity due to activation of acetylcholine receptors. The model predicted a small depolarization of the membrane potential of about 2 mV and a subsequent recovery back to the resting membrane potential, a depolarization that was well below the threshold potential for action potential firing (Figure 3C). While the observed depolarization was very small, closely resembling experimental data (Vivas et al., 2014), the model predicted a strong inhibition of KCNQ channels and substantial net PI(4,5)P_2_ depletion (Figure 3E, top and middle panels). This finding shows that our model correctly predicts that an inhibition of KCNQ channels alone is insufficient to cause action potential firing if calcium channels are not inhibited as well (Figure 3E, bottom panel). Next, we tested the predicted outcome of an inhibition of KCNMA1 channels alone. Experimental data had shown that inhibition of KCNMA1 channels without inhibition of KCNQ channels does not evoke action potential firing in rat SCG neurons (Vivas et al., 2014). We tested this by performing a 60 s long simulation of the membrane potential during which we set the conductance of KCNMA1 channels to 0 between 10 s and 50 s while keeping KCNQ channel conductance at 100%. Closely resembling experimental data, the membrane potential barely depolarized (Figure 3D). Interestingly, if we compared simulations that allowed for inhibition of L-type calcium channels by activation of muscarinic acetylcholine receptors (Figure 3F, bottom panel) which caused a strong depolarization of the membrane potential and eventually action potential firing (Figure 3A) with simulations that kept L-type channel conductance constant while activating muscarinic acetylcholine receptors (Figure 3E, bottom panel), the amounts of PI(4,5)P_2_ and KCNQ channel inhibition were identical in both simulations (Figures 3E,F, top and middle panels). These simulation results show that our model correctly predicts a simultaneous control of electrical activity of rat SCG neurons by KCNQ and KCNMA1 channels with a strong dependence of KCNQ channels on PI(4,5)P_2_ while KCNMA1 channel activity is controlled by influx of Ca^2+^ through L-type calcium channels.

**FIGURE 4:**
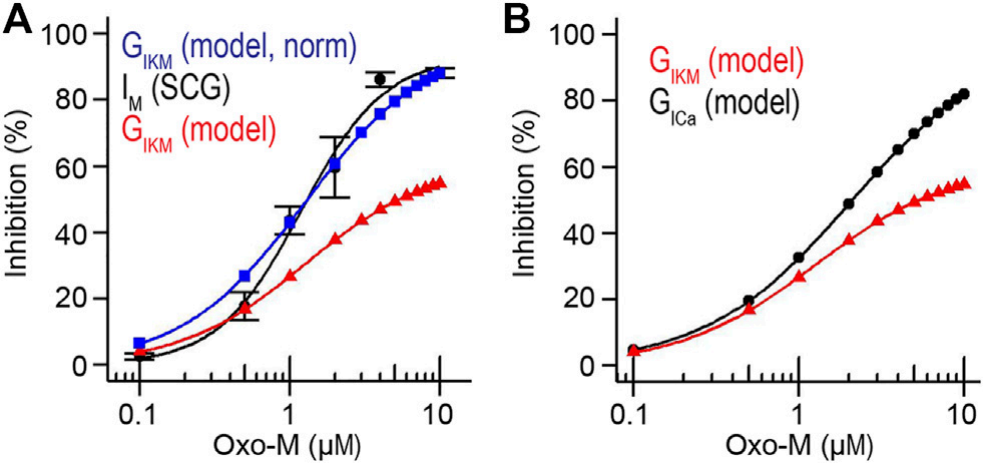
Dose-dependence of I_KM_ and I_Ca_ inhibition. **(A)**. Simulated and measured inhibition of KCNQ2/3 channel activity in response to 0.1–10.0 μM Oxo-M. Measured I_M_ data for SCG neurons taken from (Kruse et al., 2016). **(B)**. Simulated inhibition of KCNQ2/3 and L-type calcium channels in response to 0.1–10.0 μM Oxo-M.

Patch-clamp recordings on isolated rat SCG neurons had allowed for the experimental determination of an IC_50_ value of M current inhibition by Oxo-M of 1.2 ± 0.2 μM (Kruse et al., 2016). We used this value to test whether our model would provide us with a similar prediction as a way to evaluate the quality of the newly generated model. Our model predicted an IC_50_ value of 0.7 μΜ (Figure 4A), a value that is slightly lower than our experimentally determined IC_50_, however, the modelled IC_50_ is in agreement with a previously published IC_50_ value for inhibition of M current by Oxo-M in SCG neurons by Winks et al. who reported an IC_50_ of 0.7 ± 0.1 μM (Winks et al., 2005). In addition, we determined the IC_50_ for inhibition of L-type calcium channels by Oxo-M as predicted by our model and compared it with previously published data. Our model predicted an IC_50_ for L-type calcium channels of 2.1 μΜ (Figure 4B), which is in agreement with previously published IC_50_ values of 2.0 μM (Bernheim et al., 1992; Zhang et al., 2011). We concluded that our model shows a dose-dependence of KCNQ- and L-type calcium channel inhibition through activation of muscarinic acetylcholine receptors that resembles previously published experimental observations.

### Altering M_1_R Surface Density and PI(4)P 5-kinase Activity Levels as Tools to Uncouple Action Potential Firing and Intracellular Signaling Pathways

**FIGURE 5:**
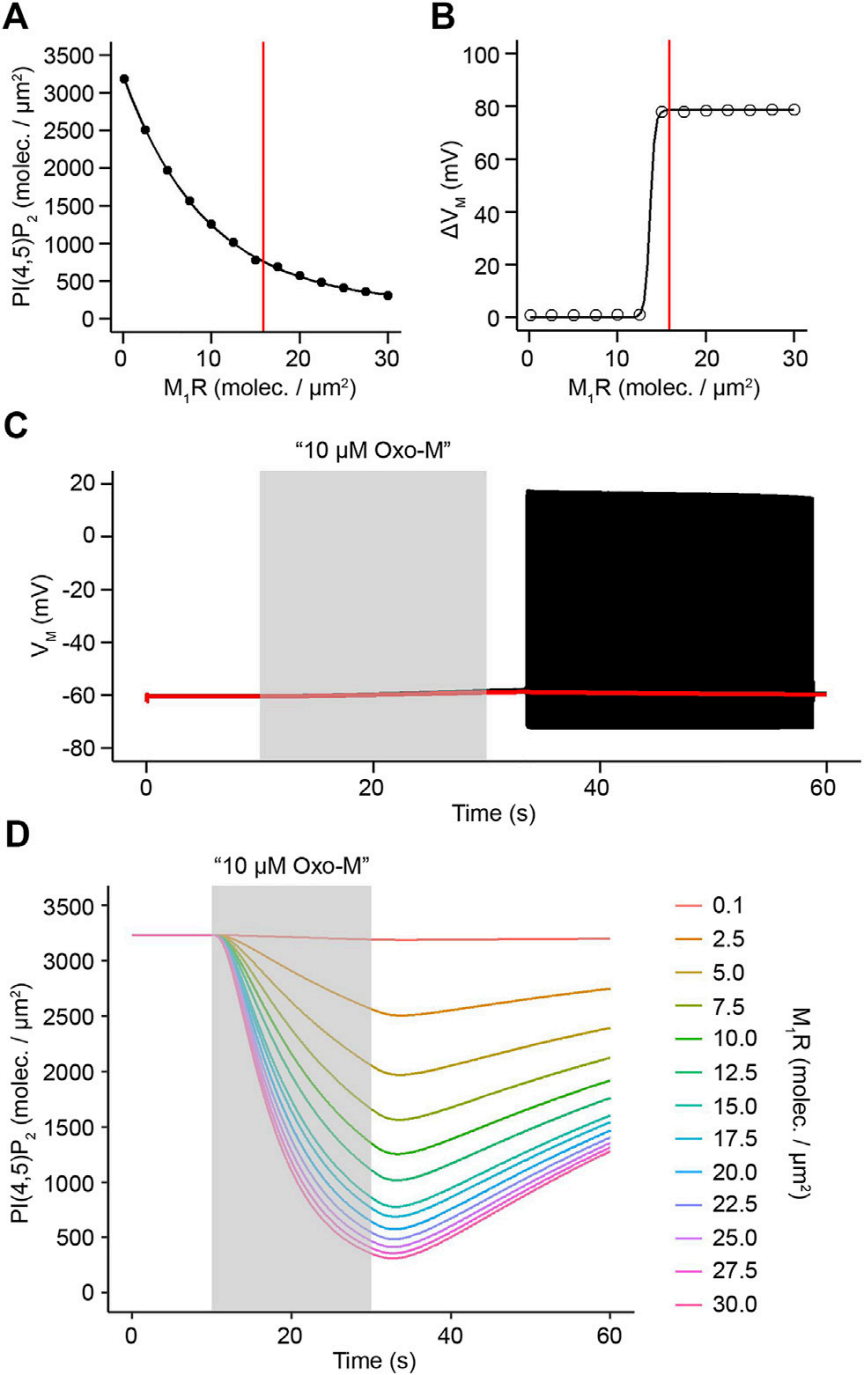
Control of PI(4,5)P_2_ depletion and action potential firing by M_1_R activity levels. **(A)**. PI(4,5)P_2_ densities after activation of varying densities of M_1_R by 10 μM Oxo-M for 20 s. Red line indicates experimentally determined M_1_R surface density of 15.87 receptors/μm^2^. **(B)**. Simulated maximum difference in membrane potential before and after activation of varying densities of M_1_R by 10 μM Oxo-M for 20 s. Red line indicates experimentally determined M_1_R surface density of 15.87 receptors/μm^2^. **(C)**. Simulated membrane potential in response to an application of 10 μM Oxo-M for 20 s at a M_1_R density of 12 receptors/μm^2^ (red trace) or a density of 16 receptors/μm^2^ (black trace). **(D)**. Time courses of PI(4,5)P_2_ levels in response to a virtual application of 10 μM Oxo-M for 20 s at indicated densities of M_1_R.

Previous biochemical analysis has shown that superior cervical ganglion neurons have a resting surface density of approximately 16 receptors/μm^2^ (Kruse et al., 2016). Receptor turnover in neuronal cells is highly dynamic though (Devreotes and Fambrough, 1975; Rudell and Ferns, 2013) and variations in receptor density are therefore likely to cause different amounts of phospholipase C activation and subsequent hydrolysis of PI(4,5)P_2_ as well as generation of second messengers such as diacylglycerol and inositol 1,4,5-trisphosphate. We used our model to develop predictions how alterations in M_1_R surface density would influence hydrolysis of PI(4,5)P_2_ and action potential generation. For this purpose, we applied a virtual stimulus of 10 μM Oxo-M for a duration of 20 s and altered M_1_R density ranging from 0 to 30 receptors/μm^2^. Increasing receptor density from 0 to 16 receptors/μm^2^ led to a sharp increase in the amount of PI(4,5)P_2_ being hydrolyzed by activation of phospholipase C, and simulations with the experimentally determined receptor density of 16 receptors/μm^2^ showed levels of ∼800 molecules/μm^2^ PI(4,5)P_2_ remaining at the end of the Oxo-M stimulation (Figure 5A), indicating a depletion of ∼ 75% of resting PI(4,5)P_2_ levels. Interestingly, increasing the receptor density further did not lead to a strong additional increase in PI(4,5)P_2_ hydrolysis, which indicates that the biochemical signaling pathway seems to reach saturation at this point and synthesis of PI(4,5)P_2_ can mostly compensate for the additional hydrolysis of PI(4,5)P_2_ by phospholipase C (Figure 5A). Next, we asked how a variation in M_1_R density influences electrical excitability of SCG neurons. We utilized the same stimulation protocol and observed a strong correlation between the ability of a stimulus to cause action potential firing and M_1_R surface density (Figure 5B). Our simulations showed that receptor densities up to 12.5 receptors/μm^2^ were insufficient to trigger action potential firing and the membrane potential showed very little depolarization upon a stimulation with 10 μM Oxo-M for 20 s (Figure 5B). Increasing the receptor density to 15 receptors/μm^2^ on the other hand caused action potential firing (Figure 5B), which indicates that this density of M_1_R seems to cause sufficient inhibition of KCNQ and L-type calcium channels if stimulated with the aforementioned application protocol. It is striking that an alteration of very few receptor molecules per μm^2^ is sufficient to seemingly function as a switch for the generation of action potential firing and that the physiological resting receptor density seems to be just above this threshold, thereby potentially allowing a SCG neuron to switch between “non-firing” and “firing” by adjusting M_1_R surface density by very few molecules (Figure 5C). Interestingly, activation of phospholipase C through surface densities below 15 receptors/μm^2^ was still capable of hydrolyzing more than 50% of PI(4,5)P_2_ (Figures 5A,D), which provides potential to generate second messengers such as DAG and IP_3_ without causing the SCG neuron to fire action potentials.

**FIGURE 6:**
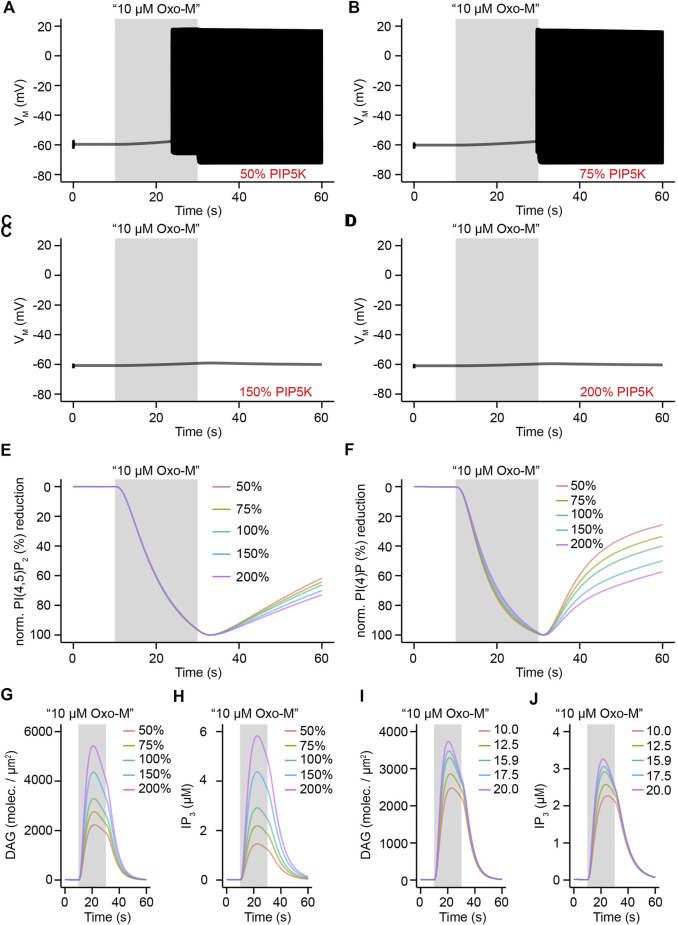
Influence of PIP5K activity on electrical activity and second messenger generation in rat SCG neurons. **(A)**. Simulated membrane potential in response to an application of 10 μM Oxo-M for 20 s under conditions of reduction of PIP5K activity by 50%. **(B) – (D)**. Same as in A, but under conditions of 75, 150, and 200% activity of PIP5K, respectively. **(E) – (H)**. Normalized reductions or membrane densities and concentrations for levels of PI(4,5)P_2_ **(E)**, PI(4)P **(F)**, diacylglycerol **(G)**, and inositol 1,4,5-trisphosphate **(H)** in response to an application of 10 μM Oxo-M for 20 s with indicated levels of PIP5K activity. **(I)** – **(J)**. Same as in G and H, but for indicated levels of M_1_R surface density (molecules/μm^2^) under conditions of 100% PIP5K activity.

We hypothesized that if small alterations of M_1_R surface density are sufficient to modify phospholipase C activation to an extent that allows for switching between a “firing” and “non-firing” mode of electrical activity, an alteration in PI(4,5)P_2_ synthesis pathways might have a similar effect and provide an equivalent level of control over electrical activity of SCG neurons. We tested this hypothesis by either reducing PI(4)P 5-kinase activity in our model to levels of 50 and 75% of original activity or by increasing it to 150 or 200% of its original activity while keeping M_1_R density at 16 molecules/μm^2^. Given that PI(4)P 5-kinases catalyze the last reaction step in the synthesis of PI(4,5)P_2_ from phosphatidylinositol (PI) (Figure 9B) we considered them a reasonable biochemical target and a potential modulator of electrical activity (Di Paolo et al., 2004). In agreement with our hypothesis, a virtual application of 10 μM Oxo-M for 20 s under conditions of reduced PI(4)P 5-kinase activity caused increased action potential firing compared to control conditions (Figures 6A,B, control data shown in Figure 5C, black trace). Increasing PI(4)P 5-kinase activity to 150 or 200% of its original activity on the other hand rendered the stimulus insufficient to evoke action potential firing and led to only a small depolarization of the membrane potential (Figures 6C,D). These simulation results support our hypothesis that small modifications of PI(4)P 5-kinase activity can serve as “switches” in the same way as a modification of M_1_R surface density can do. This switch-like behavior is not caused by differences in the speed at which PI(4,5)P_2_ and PI(4)P are hydrolyzed by phospholipase C as can be seen by comparing normalized traces of PI(4,5)P_2_ and PI(4)P depletion under conditions of 50, 75, 100, 150, or 200% of PI(4)P 5-kinase activity, respectively (Figures 6E,F). A critical property of reductions of either M_1_R surface density or PI(4)P 5-kinase activity is the ability of SCG neurons to still produce significant amounts of important second messengers that are generated as products of PLC-mediated PI(4,5)P_2_ hydrolysis. Simulations with 50 or 75% reduced PI(4)P 5-kinase activities showed levels of DAG or IP_3_ that were still sufficient to trigger the activation of protein kinases in the case of DAG or cause release of calcium from intracellular calcium stores in the case of IP_3_ (Figures 6G,H). Similarly, varying the surface density of M_1_R from 10 to 20 molecules/μm^2^ showed significant production of DAG and IP_3_ under all tested conditions (Figures 6I,J). These results indicate that small variations in the surface density of M_1_R or PI(4)P 5-kinase activity are predicted to lead to a switch between firing and non-firing of action potentials while the production of second messengers such as DAG and IP_3_ follows a more gradual progression under these conditions. A reduction of M_1_R surface density or PI(4)P 5-kinase activity could therefore serve as an aforementioned switch that uncouples electrical activity from intracellular signaling pathways and allows SCG neurons to utilize PLC-mediated hydrolysis of PI(4,5)P_2_ to generate second messenger molecules without evoking action potential firing.

### Influence of PI(4,5)P_2_ Levels on Electrical Excitability of SCG Neurons in Response to Varying Levels of PI(4)P 5-Kinase Activity

**FIGURE 7:**
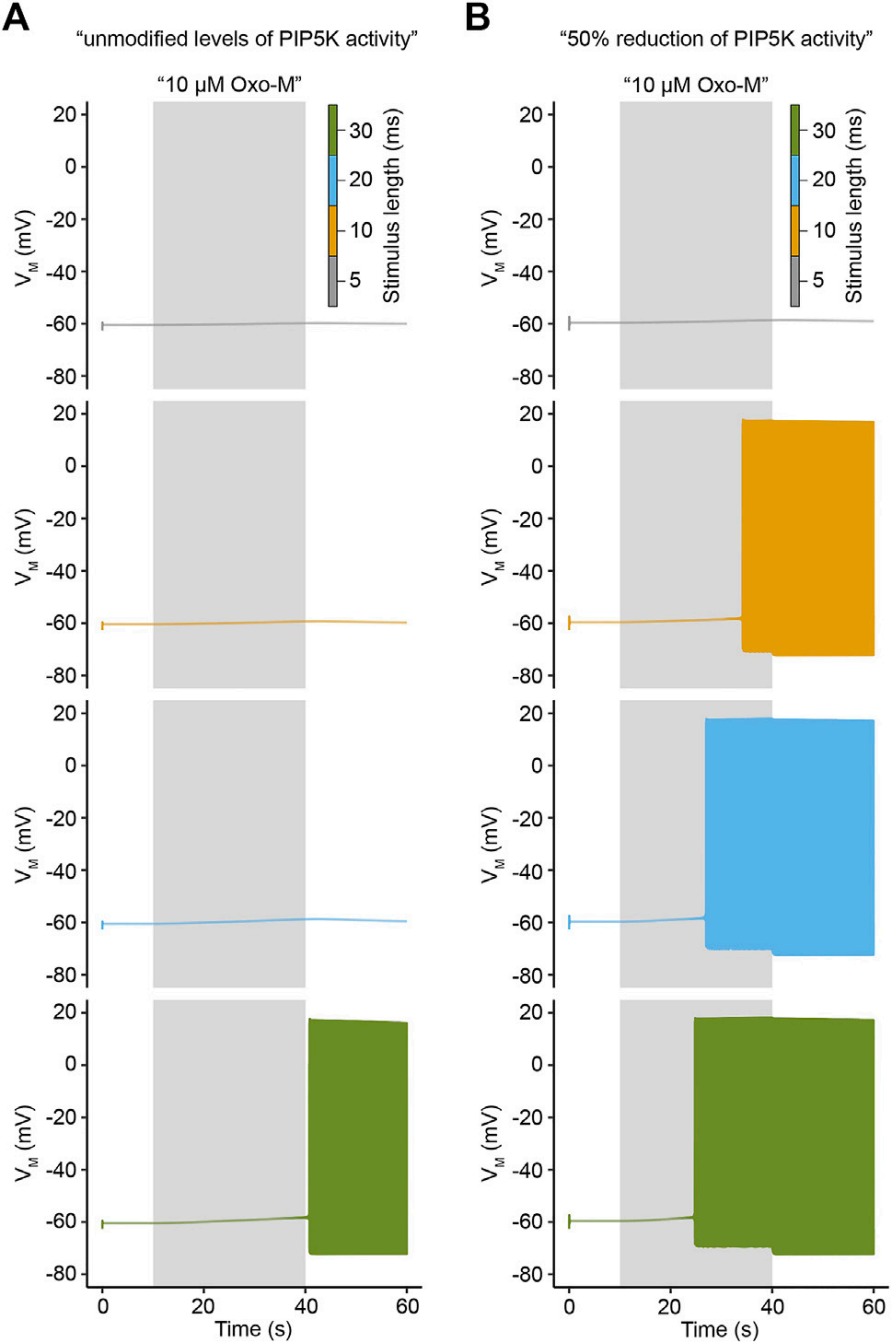
Influence of stimulus duration on electrical activity under varying conditions of PIP5K activity. **(A)**. Simulated membrane potentials in response to a 30 s application of 10 μM Oxo-M for indicated stimulus lengths at a stimulation frequency of 10 Hz under conditions of unmodified PIP5K activity. **(B)**. Same as in A, but for 50% reduction of PIP5K activity.

Activation of M_1_R by Oxo-M in a laboratory setting is usually accomplished by prolonged continuous application of the agonist, e.g. continuous stimulation for 20–30 s (Suh and Hille, 2002; Dickson et al., 2016; Kruse et al., 2016). While these stimulation patterns are well suited for a variety of experiments, they do not reproduce physiological situations in which acetylcholine is present for only a few milliseconds, but might stimulate a neuron several times per second at frequencies of 2–10 Hz (Brown and Selyanko, 1985; Ivanov and Purves, 1989). Such stimulation frequencies are difficult to reproduce experimentally, so we decided to apply such stimulation patterns to our model to analyze how PI(4,5)P_2_ levels and electrical activity would develop under such conditions. For this purpose, we applied virtual stimuli of 10 μM Oxo-M at a frequency of 10 Hz for a duration of 30 s and varied the length of an individual stimulus from 5 to 30 ms. We first focused on electrical activity and observed that under conditions of unmodified PI(4)P 5-kinase activity the membrane potential reacted with only small subthreshold depolarizations to individual stimuli that were 5, 10, or 20 ms long. However, a duration of 30 ms for an individual application of 10 μΜ Oxo-M evoked action potential firing (Figure 7A). Next, we reduced PI(4)P 5-kinase activity by 50% and repeated our virtual experiment. Under these conditions, individual stimuli as short as 10 ms evoked action potential firing, indicating a strongly increased electrical excitability of the neurons (Figure 7B).

**FIGURE 8:**
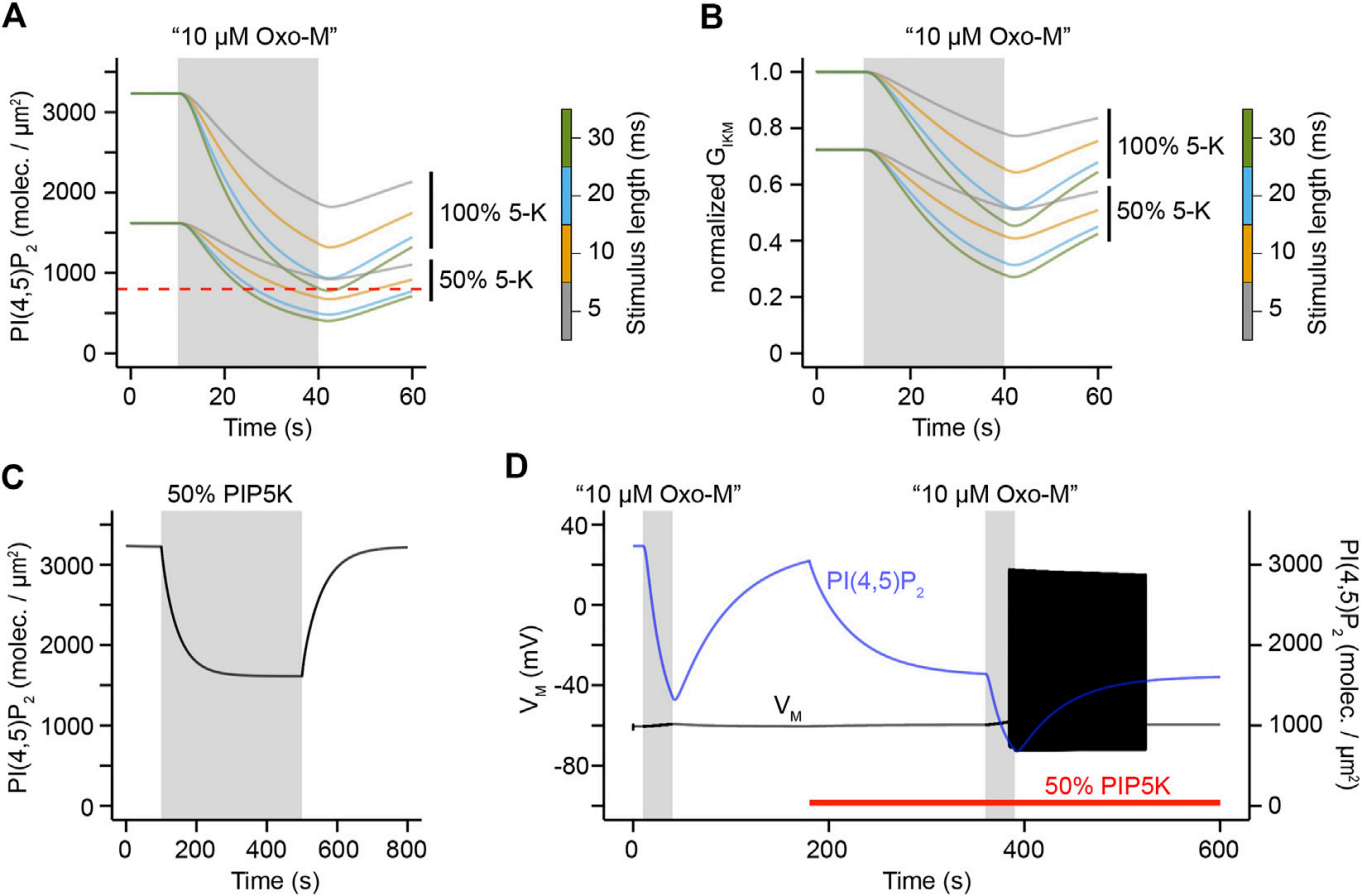
Relationship of PIP5K activity, PI(4,5)P_2_ levels, and electrical excitability. A. Simulations of PI(4,5)P_2_ densities in response to a 30 s application of 10 μM Oxo-M for indicated levels of PIP5K activity and stimulus durations. All stimulations were simulated for a frequency of 10 Hz. **(B)**. Same as **(A)**, but for KCNQ channel conductance. **(C).** Simulation of PI(4,5)P_2_ levels under conditions of unmodified PIP5K activity (white background) or reduction of PIP5K activity by 50% (gray background). **(D).** Simulation of membrane potential and PI(4,5)P_2_ density in response to 30 s applications of 10 μM Oxo-M at a stimulus frequency of 10 Hz and a stimulus duration of 10 ms. Red bar indicates simulation period with a 50% reduction of PIP5K activity.

Could this increased excitability under conditions of reduced PI(4)P 5-kinase activity be caused by PI(4,5)P_2_ levels that are prior to activation of M_1_R already only slightly above the threshold for decreasing KCNQ channel sufficiently to evoke action potential firing? We addressed this question by analyzing PI(4,5)P_2_ levels for conditions of unmodified and reduced PI(4)P 5-kinase levels. Interestingly, before activation of M_1_R we observed PI(4,5)P_2_ levels that are above a density of ∼ 800 molecules/μm^2^ (Figure 8A) which we had determined to be approximately the threshold for enough reduction of KCNQ channel activity to evoke action potential firing (Figure 5A). It is important to note though that under conditions of reduced PI(4)P 5-kinase activity the observed PI(4,5)P_2_ density is only twice the threshold density while the PI(4,5)P_2_ density under control conditions is four times larger than the threshold density (Figure 8A). Under control conditions, stimulation with the aforementioned duration times at a frequency of 10 Hz causes only the PI(4,5)P_2_ curve for a stimulation of 30 ms length of an individual pulse to drop below this threshold, but the curves for shorter stimulus durations remain above it (Figure 8A). This is in line with the observed firing behavior for unmodified levels of PI(4)P 5-kinase activity (Figure 7A). On the other hand, the PI(4,5)P_2_ curves under conditions of reduced PI(4)P 5-kinase activity all drop below this threshold except for the curve for a 5 ms long stimulation (Figure 8A), again following exactly the observed pattern of electrical activity (Figure 7B). Similarly, levels of M current conductance followed the same pattern (Figure 8B) and allowed us to conclude that reduced PI(4,5)P_2_ levels due to a reduction of PI(4)P 5-kinase activity seemingly render SCG neurons significantly more excitable.

These findings led us to the hypothesis that transient reductions in PI(4)P 5-kinase activity could be a regulatory switch for SCG neurons that would allow them to flip back and forth between responding to a stimulus with action potential firing and activation of intracellular signaling pathways that depend on DAG and IP_3_, or to remain electrically silent, but allow for second messenger generation. This hypothesis requires that adjustments in PI(4,5)P_2_ levels can happen quickly enough to make this switch-like behavior physiologically reasonable for a neuron. We tested this hypothesis by simulating PI(4,5)P_2_ levels over a time period of 800 s during which we reduced PI(4)P 5-kinase activity levels by 50% for the time period of 180 s–480 s. Interestingly, our simulations showed that such transient switches in activity for very few minutes are sufficient to equilibrate PI(4,5)P_2_ to a new steady-state (Figure 8C), thereby allowing for the cell to quickly adjust resting PI(4,5)P_2_ levels within short time periods. Next, we asked whether such an adjustment of PI(4)P 5-kinase activity would indeed produce the aforementioned patterns of electrical activity. For this purpose, we simulated both PI(4,5)P_2_ and electrical activity over a time period of 600 s and kept PI(4)P 5-kinase activity at its control level for the first 180 s. Afterwards, we reduced its activity by 50% for the rest of the simulation (red bar in Figure 8D). In this simulation, we applied virtual stimuli of 10 μΜ Oxo-M at a frequency of 10 Hz for 10 ms durations between 10 and 40 s of the simulation and again between 360 and 390 s (Figure 8D). In line with our hypothesis, the first stimulation which occurred under conditions of unmodified PI(4)P 5-kinase activity decreased PI(4,5)P_2_ levels and generated second messengers, but failed to depolarize the neuron sufficiently to evoke action potential firing (Figure 8D). The second stimulus though which was applied under conditions of reduced PI(4)P 5-kinase activity and after equilibration of PI(4,5)P_2_ densities to a new and reduced steady-state caused not only PI(4,5)P_2_ hydrolysis, but evoked action potential firing (Figure 8D). These observations support the hypothesis that transient alterations in PI(4)P 5-kinase activity could serve as a switch for neurons to temporarily alter between stimulus responses that combine second messenger generation with action potential firing or ones that evoke only second messenger generation.

### Simulation of L-type Channel Activity Shows Activation near Resting Membrane Potential

**FIGURE 9:**
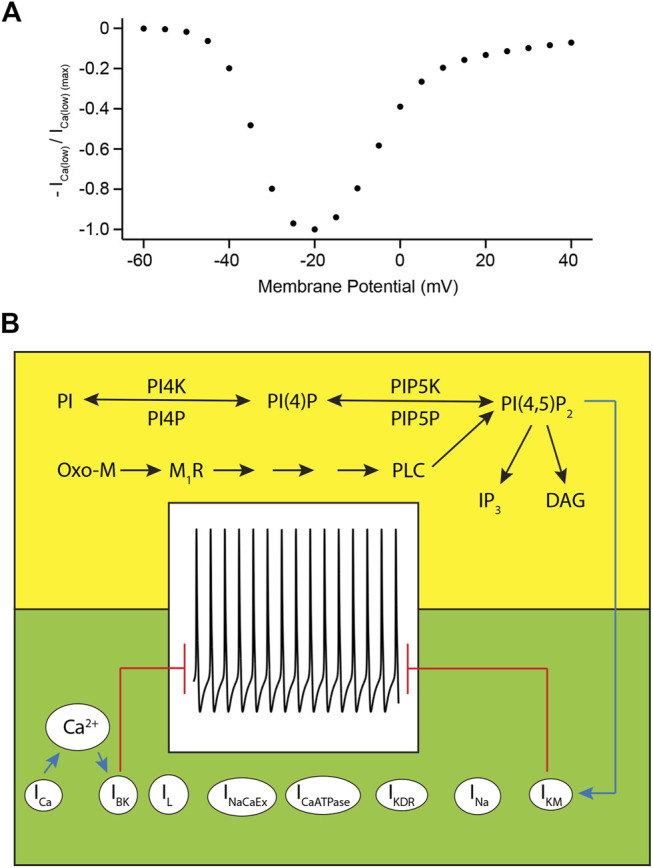
Voltage-dependence of low-threshold L-type calcium channels and model characteristics. **(A)**. Normalized simulated current amplitude of low-threshold L-type calcium channels plotted against membrane potential. **(B)**. Illustration of regulation of action potential firing of rat SCG neurons by phosphoinositide metabolism (yellow background) and ion channel activity (green background). Blue arrows indicate activation of KCNQ2/3 channels by PI(4,5)P_2_ or of KCNMA1 channels by Ca^2+^ ions entering the cell through L-type calcium channels. Red lines indicate that non-inhibited activity of KCNQ2/3 and KCNMA1 channels prevents action potential firing.

Activation of KCNMA1 channels through Ca^2+^-influx mediated by L-type calcium channels requires these channels to be active at membrane potentials near the resting membrane potential. Electrophysiological recordings of KCNMA1 channels co-expressed with Ca_V_1.3 channels have provided evidence that co-expression of these channels shifts their activation thresholds to membrane potentials around -50 mV (Vivas et al., 2017) which is well in line with membrane potential depolarizations evoked by closure of KCNQ channels upon muscarinic stimulation (Vivas et al., 2014). Simulations of I/V curves of low-threshold calcium channels in our model show activation of these channels around a membrane potential of -50 mV (Figure 9A), thereby reproducing experimental results obtained by our other groups (Vivas et al., 2017) and providing an explanation for the experimentally observed dependence of KCNMA1 channels on L-type calcium channel activity at membrane potentials near the resting membrane potential.

## Discussion

### Validation of Model

Generation of action potential firing in superior cervical ganglion neurons has been shown to be controlled by several different factors. First, voltage-gated potassium channels such as KCNQ and KCNMA1 channels conduct a potassium efflux that stabilizes the resting membrane potential, thereby requiring an inhibition of both channels to allow for the generation of action potential firing (Suh and Hille, 2002; Zaika et al., 2006; Brown et al., 2007; Vivas et al., 2014). Both KCNQ and KCNMA1 channels are also ligand-activated, with KCNQ channels requiring interaction with PI(4,5)P_2_ and KCNMA1 channels with Ca^2+^ (Suh and Hille, 2002; Vivas et al., 2017). Our model reproduces these dependencies, and action potential firing is only evoked in the model if both KCNQ and KCNMA1 channels are inhibited due to a loss of interaction with PI(4,5)P_2_ and Ca^2+^, respectively. Second, current-clamp recordings on SCG neurons have shown that stimulation of SCG neurons with Oxo-M at supramaximal concentrations leads to a slow (20–40 s) sub-threshold depolarization before the membrane potential reached the threshold for activation of voltage-gated sodium channels (Vivas et al., 2014). This slow depolarization has been explained by ongoing net depletion of PI(4,5)P_2_ that causes an accumulation of deactivated KCNQ channels (Suh and Hille, 2002). Our newly developed model recapitulates this important property (see Figure 3C for an example of this slow depolarization) and places action potential firing under the control of PI(4,5)P_2_ if Ca^2+^ levels are sufficiently low to deactivate KCNMA1 channels. Third, Ca^2+^ needed for activation of KCNMA1 channels enters SCG neurons through L-type Ca^2+^ channels that are located in close proximity of KCNMA1 channels and is not provided by release of Ca^2+^ from intracellular stores (Vivas et al., 2014; Vivas et al., 2017). The model reproduces this interplay between L-type Ca^2+^ channel and KCNMA1 channel activity and places the activity of L-type Ca^2+^ channels under the control of muscarinic acetylcholine receptors. In addition, our model reproduces depolarization as well as repolarization kinetics as observed by perforated patch-clamp recordings from rat SCG neurons, and correctly simulates peak amplitudes and firing frequencies of action potentials evoked by activation of muscarinic acetylcholine receptors (Vivas et al., 2014). In conclusion, the newly generated model resembles all known characteristics of experimentally observed action potential firing of rat SCG neurons and combines this description of electrical activity with a previously published quantitative simulation of phosphoinositide metabolism of these neurons (Kruse et al., 2016), therefore allowing for a simulation of electrical activity of rat SCG neurons under the control of muscarinic acetylcholine receptors and phosphoinositide metabolism.

### Regulation of Action Potential Firing by Surface Density of Muscarinic Acetylcholine Receptors and PI(4,5)P_2_ Synthesis Kinetics

Our simulations showed that small variations in the surface density of muscarinic acetylcholine receptors or kinetics of PI(4,5)P_2_ synthesis changed electrical excitability of rat SCG neurons significantly. Are such modulations of surface density and PI(4,5)P_2_ synthesis kinetics plausible and if so, what physiological consequences does such a modulation have? We first turn our attention to mechanisms that govern surface density of muscarinic acetylcholine receptors. Several different studies have provided evidence that surface localization of muscarinic acetylcholine receptors is neither uniform nor stable in different kinds of neurons, but can change in a short amount of time based on the physiological situation of the specific neuron. Bernard et al. showed that the localization of M_4_R in striatal neurons depends on the cholinergic environment and that stimulation of these neurons with oxotremorine triggers translocation of M_4_R from the cell surface to endosomes (Bernard et al., 1999). In addition, several groups have shown that PDZ domain-containing proteins such as LARG, RGS3, PDZ-RhoGEF, and Spinophilin function as regulators of trafficking and surface density of practically all different types of muscarinic acetylcholine receptors (Dunn and Ferguson, 2015). In addition to these mechanisms, proteins such as receptor-activity-modifying-proteins (RAMPs) and RACK1 (Receptor for Activated C-Kinase 1) have been linked to regulation of cell surface density of several different types of GPCRs (Achour et al., 2008). While this list is far from being complete it highlights that GPCR cell surface density has been shown to be dynamic and controlled by a variety of different mechanisms.

Similar to rapid changes in cell surface receptor density, kinetics of PI(4,5)P_2_ synthesis have been shown to be dynamic and adjustable to the specific needs of a cell in a given physiological situation. One example of such a transient adjustment of PI(4,5)P_2_ synthesis kinetics is activation of the Wnt3a pathway. Qin et al. showed that activation of the cell surface receptor Frizzled followed by recruitment of the cytoplasmic protein Dishevelled led to stimulation of PI(4,5)P_2_ synthesis via activation of both phosphatidylinositol 4-kinase (PI4K) and PI(4)P 5-kinases (Pan et al., 2008; Qin et al., 2009). The interaction between Dishevelled and phosphatidylinositol 4-phosphate 5-kinase seems to be mediated by the DIX domain of Dishevelled, which allows for a rapid and transient control of phosphatidylinositol 4-phosphate 5-kinase activity (Hu et al., 2015). The lipid environment of the plasma membrane itself has been shown to be another regulator of phosphatidylinositol 4-phosphate 5-kinase activity. Recent work by Nishimura et al. showed that Osh proteins generate nanodomains in the plasma membrane that are enriched in unsaturated phosphatidylserine and sterols which synergistically stimulate phosphatidylinositol 4-phosphate 5-kinase activity (Nishimura et al., 2019). Small variations in Osh protein levels can alter this microenvironment and lead to alterations of PI(4,5)P_2_ levels. Lastly, protein levels of PIP5Ks are dynamic and alterations of PIP5K amounts or of proteins that activate PIP5Ks such as Ras associated domain family 4 (RASSF4) have been shown to change cellular phosphoinositide levels (de la Cruz et al., 2020). It should be noted that kinetics of PI(4,5)P_2_ synthesis are not only controlled by activity levels of phosphatidylinositol 4- and 5-kinases and phosphatidylinositol 4,5-bisphosphate 5-phosphatases as well as by phosphatidylinositol 4-phosphate phosphatases, but also by levels of substrates such as phosphatidylinositol and phosphatidylinositol 4-phosphate (Dai et al., 2016). In conclusion, transient adjustments of PI(4,5)P_2_ synthesis kinetics have been shown for various different cell types and mechanisms, and make it likely that such dynamic adjustments of PI(4,5)P_2_ levels can be used by neurons and other cell types to regulate their activity and responses to stimuli that activate pathways which involve phosphoinositide signaling.

What are the potential benefits of adjustments of cell surface receptor density and PI(4,5)P_2_ levels for a SCG neuron? Our simulations show that relatively modest alterations in either of these parameters can act like a switch for the SCG neuron that either triggers action potential firing in response to a stimulation with acetylcholine, or prevents it. We showed that this switch-like behavior can uncouple the generation of the second messengers inositol 1,4,5-trisphosphate and diacylglycerol from generation of action potential firing, thereby allowing the SCG neuron to use muscarinic acetylcholine receptors either only for the activation of intracellular signaling pathways or for a dual generation of electrical activity and second messenger molecules. In addition, these adjustments allow for a dosed response to acetylcholine and can transiently alter the sensitivity of a neuron to an incoming stimulus, thereby providing flexibility for the neuron receiving the stimulus. Achieving this level of flexibility with adjustments of just two parameters while using only one signaling system provides robustness and reduces the need for the cell to provide separate signaling systems for different physiological needs and conditions.

### Analysis of PI(4,5)P_2_ Dependence of KCNMA1 Channels in SCG Neurons

Our expression analysis showed that KCNMA1 channels in rat SCG neurons are composed of only α-subunits. Given both previous reports that any PI(4,5)P_2_ dependence of KCNMA1 channels is caused by interaction of KCNMA1 α-subunits with β-subunits (Hille et al., 2015) and our own correlating finding that KCNMA1 channel α-subunits do not show alterations of their activity by net PI(4,5)P_2_ depletion, the question can be raised what physiological advantage it could have for SCG neurons to control action potential firing through two potassium channels that are regulated by different ligands? The answer to this question might be the generation of a control system that provides enhanced flexibility for the neuron. Placing both potassium channels under the control of PI(4,5)P_2_ would lead to a situation in which net depletion of PI(4,5)P_2_ almost always causes action potential generation. Placing KCNQ potassium channels under the control of PI(4,5)P_2_ and KCNMA1 α-subunits under the control of intracellular Ca^2+^ levels though allow for a SCG neuron to control whether a signal evokes both electrical activity and second messenger generation or only activation of intracellular signaling pathways. Under these conditions, receptor activation can cause net depletion of PI(4,5)P_2_ that deactivates KCNQ channels, however, if L-type Ca^2+^ channels are not inhibited simultaneously, KCNMA1 channels would remain active and prevent action potential firing, therefore allowing for activation of intracellular signaling pathways in response to muscarinic stimulation without evoking action potential firing. A simultaneous depletion of PI(4,5)P_2_ and inhibition of L-type Ca^2+^ channels would inhibit both KCNQ and KCNMA1 channels though, and cause action potential firing of the SCG neuron.

### Future Uses and Expansion of Model

Our model provides a kinetic description of electrical activity and phosphoinositide metabolism in rat superior cervical ganglion neurons; however, it does not contain spatial information. Currently, phosphoinositide metabolism is mathematically described as a series of biochemical reactions in one compartment and it does not simulate the synthesis of phosphoinositides in different compartments and the transport processes of these phospholipids between different intracellular organelles (Saheki and De Camilli, 2017) (Figure 9B). It would be of great interest to expand the model to include such spatial information. Recent work by Zewe et al. and Pemberton et al. using newly developed fluorescent biosensors for the most prominent member of the phosphoinositide family, phosphatidylinositol (PI), revealed a very low presence of PI at the plasma membrane, but provided evidence for pools of PI in the endoplasmic reticulum, cytosolic leaflets of the Golgi complex, peroxisomes, and the outer mitochondrial membrane (Pemberton et al., 2020; Zewe et al., 2020). These new fluorescent biosensors will allow us to not only acquire experimental data needed for the simulation of compartmental transport of PI, but also provide the possibility to determine kinetics of PI synthesis and breakdown, thereby significantly enhancing the mathematical description of phosphoinositide metabolism in our current model. The addition of spatial information to the model would also allow to describe the interplay of KCNMA1 and L-type calcium channels in a stochastic manner as previously described by Cox (Cox, 2014). Lastly, it would be of great interest to expand the description of regulation of L-type calcium channel activity in our model. Work by Suh et al. has shown that the association of certain β-subunits with α-subunits of L-type calcium channels renders them partly sensitive to PI(4,5)P_2_ levels (Suh et al., 2012), and Liu et al. have shown that arachidonic acid can serve as a modulator of L-type calcium channel activity (Liu et al., 2006). Currently, our model uses a simplified approach to calculate L-type calcium channel inhibition by multiplying the maximum conductance of L-type calcium channels with the ratio of activated muscarinic acetylcholine receptors divided by the total number of receptors. This calculation does not take the aforementioned regulatory mechanisms of L-type calcium channels into account, but gaining more information about the molecular nature of regulation of these channels in SCG neurons would allow for a more detailed mathematical description of their activity in a model. An inclusion of these regulatory mechanisms would therefore provide an opportunity to use such an expanded version of our model for testing of more detailed hypotheses regarding the regulation of action potential firing by phosphoinositide metabolism.

The model presented here consists of two components, the first one being a description of phosphoinositide metabolism and muscarinic acetylcholine receptor type I signaling in rat SCG neurons (highlighted in the yellow box in Figure 9B), and the second component being a mathematical description of ion channel activities in rat SCG neurons which are responsible for action potential firing in the neurons (highlighted in the green box in Figure 9B). As illustrated by the background color coding in Figure 9B, these two components of the model can be treated as two separate mathematical descriptions, but they are linked via the regulation of KCNQ channel activity through PI(4,5)P_2_ (indicated by blue arrow in Figure 9B). Mathematically, this relationship is provided by Eqs. (16, 17) listed in the Results section. As can be seen from this equation, our model assumes an interaction of one PI(4,5)P_2_ molecule per KCNQ channel subunit. This is in contrast to the results published by Falkenburger et al. (Falkenburger et al., 2010b) which suggested an interaction of more than one molecule PI(4,5)P_2_ per channel subunit. Our decision to simulate this interaction with just one molecule PI(4,5)P_2_ per channel subunit is based on 1) a better reproduction of experimental data, e.g. kinetics of membrane potential depolarization after activation of M_1_R and duration of action potential firing upon PI(4,5)P_2_ depletion, compared to simulations that assumed the interaction of more than molecule PI(4,5)P_2_ per KCNQ channel subunit, and 2) recently published data by Sun and MacKinnon that analyzed the structural basis of human KCNQ1 channel modulation and gating by PI(4,5)P_2_ (Sun and MacKinnon, 2020). Their work showed the interaction of just one molecule PI(4,5)P_2_ with KCNQ1 channels in a region of the protein that is highly conserved among KCNQ channels. Sun and MacKinnon concluded that their data provides evidence that the findings obtained from KCNQ1 channels apply to other members of the KCNQ channel family as well. This structural analysis of KCNQ channel interaction with PI(4,5)P_2_ supported our observation that simulations assuming the interaction of only one molecule PI(4,5)P_2_ per KCNQ channel subunit resulted in a better reproduction of previously published experimental data (Vivas et al., 2014).

As mentioned before, L-type calcium activity is regulated in a complex manner that involves a small, diffusible second messenger signaling pathway as described by Mathie et al. (Mathie et al., 1992), but our model does not include such signaling due to a lack of a more detailed molecular characterization of this pathway. A mathematical description of this pathway would result in a second molecular link between the model of muscarinic signaling in the top part of Figure 9B and L-type calcium channel activity (depicted as “I_Ca_” in Figure 9B), thereby indicating how signaling through muscarinic acetylcholine signaling regulates both KCNQ- and KCNMA1 channel activity.

In conclusion, our model allowed us to identify small alterations of surface density of muscarinic acetylcholine receptors type I or changes of PI(4)P 5-kinase activity as potential molecular switches that can uncouple the generation of second messengers such as diacylglycerol and inositol 1,4,5-trisphosphate from action potential firing. Previous work from our group and others have identified PI 4-kinases and lipid transfer processes as molecular targets that can alter their activity depended on the physiological needs of a cell (Kruse et al., 2016; Dickson, 2019; Myeong et al., 2020). Our model provides a framework that allows for an analysis of the predicted outcomes of alterations in these pathways, thereby providing a toolkit that can aid not only in the development of new hypotheses and the design of targeted experiments to gain a better understanding of the connection of phosphoinositide metabolism and electrical activity of SCG neurons, but also provide further insight into the mechanisms governing the regulation of activity of sympathetic neurons in health and disease.

## Data Availability

Publicly available datasets were analyzed in this study. This data can be found here: https://github.com/Martin-Kruse/SCG-PI-excitability.

## Abbreviations

ATP, adenosine triphosphate; M_1_R, muscarinic acetylcholine receptor type I; Oxo-M, oxotremorine methiodide; PI, phosphatidylinositol; PI(4)P, phosphatidylinositol 4-phosphate; PI(4,5)P_2_, phosphatidylinositol 4,5-bisphosphate; PI4K, phosphatidylinositol 4-kinase; PIP5K, phosphatidylinositol 4-phosphate 5-kinase; PLC, phospholipase C; RACK1, receptor for Activated C-Kinase 1; RAMP, receptor-activity-modifying-protein; SCG, superior cervical ganglion; VSP, voltage-sensing phosphatase.
