# Supplementary material for: Control of Neuronal Excitability by Cell Surface Receptor Density and Phosphoinositide Metabolism
Source: Front Pharmacol. 2021 Apr 21;12:663840. doi: 10.3389/fphar.2021.663840 (PMC8097148; doi:10.3389/fphar.2021.663840)
Supplement: Supplementary file 1 [file datasheet1.docx]

**Supplemental Information: Description of ion channel activities**

The individual equations for KCNQ channels were written as the following:

$$\begin{aligned} \alpha_{KM}=\exp\left( \frac{\left( 0.001\cdot{}_{KM}\cdot\left( V-V_{halfKM} \right)\cdot96400 \right)}{(8.315\cdot T)} \right)\#\left( 1 \right) \end{aligned}$$

$$\begin{aligned} \beta_{KM}= \exp\left( \frac{\left( 0.001\cdot{}_{KM}\cdot gm_{KM}\cdot\left( V-V_{halfKM} \right)\cdot96400 \right)}{(8.315\cdot T)} \right) \#\left( 2 \right) \end{aligned}$$

$$\begin{aligned} k_{\infty}=\frac{1}{1+ \alpha_{KM}\left( V \right)}\#\left( 3 \right) \end{aligned}$$

$$\begin{aligned} \tau_{KM}=\frac{\left( \frac{\beta_{KM}\left( V \right)}{q{10}_{KM}\cdot a0_{KM}\cdot\left( 1+ \alpha_{KM}\left( V \right) \right)} \right)}{1000} \#\left( 4 \right) \end{aligned}$$

The equations for KCNMA1 channels were written as the following:

$$\begin{aligned} \alpha_{BK}=\frac{\left( \left( \frac{Ca_{C}}{1000} \right)\cdot abar \right)}{\left( \left( \frac{Ca_{C}}{1000} \right)+\left( k1\cdot\exp\left( -2\cdot d1\cdot F\cdot\frac{\frac{V}{R}}{T} \right) \right) \right)}\#\left( 5 \right) \end{aligned}$$

$$\begin{aligned} \beta_{BK}=\frac{bbar}{1+\frac{\left( \frac{Ca_{C}}{1000} \right)}{k2\cdot\exp\left( -2\cdot d2\cdot F\cdot\frac{\frac{V}{R}}{T} \right)}}\#\left( 6 \right) \end{aligned}$$

$$\begin{aligned} o_{\infty}=\frac{\alpha_{BK}\left( Ca_{C}, V \right)}{\alpha_{BK}\left( Ca_{C},V \right)+ \beta_{BK}\left( Ca_{C},V \right)}\#\left( 7 \right) \end{aligned}$$

$$\begin{aligned} \tau_{BK}=\frac{\left( \frac{1}{\alpha_{BK}\left( Ca_{C},V \right)+ \beta_{BK}\left( Ca_{C},V \right)} \right)}{1000}\#\left( 8 \right) \end{aligned}$$

All equations for KDR channels were written as the following:

$$\begin{aligned} \alpha_{n}= \exp\left( \frac{\left( 0.001\cdot{}_{n}\cdot\left( V-V_{halfn} \right)\cdot96400 \right)}{(8.315\cdot T)} \right)\#\left( 9 \right) \end{aligned}$$

$$\begin{aligned} \beta_{n}= \exp\left( \frac{\left( 0.001\cdot{}_{n}\cdot gm_{n}\cdot\left( V-V_{halfn} \right)\cdot96400 \right)}{(8.315\cdot T)} \right)\#\left( 10 \right) \end{aligned}$$

$$\begin{aligned} n_{\infty}= \frac{1}{1+ \alpha_{n}\left( V \right)}\#\left( 11 \right) \end{aligned}$$

$$\begin{aligned} \tau_{n}= \frac{\left( \frac{\beta_{n}\left( V \right)}{q10\cdot a0_{n}\cdot\left( 1+ \alpha_{n}\left( V \right) \right)} \right)}{1000}\#\left( 12 \right) \end{aligned}$$

$$\begin{aligned} \alpha_{l}= \exp\left( \frac{\left( 0.001\cdot{}_{l}\cdot\left( V-V_{halfl} \right)\cdot96400 \right)}{(8.315\cdot T)} \right)\#\left( 13 \right) \end{aligned}$$

$$\begin{aligned} \beta_{l}= \exp\left( \frac{\left( 0.001\cdot{}_{l}\cdot gm_{l}\cdot\left( V-V_{halfl} \right)\cdot96400 \right)}{(8.315\cdot T)} \right)\#\left( 14 \right) \end{aligned}$$

$$\begin{aligned} l_{\infty}= \frac{1}{1+ \alpha_{l}\left( V \right)}\#\left( 15 \right) \end{aligned}$$

$$\begin{aligned} \tau_{l}= \frac{\left( \frac{\beta_{l}\left( V \right)}{q10\cdot a0_{l}\cdot\left( 1+ \alpha_{l}\left( V \right) \right)} \right)}{1000}\#\left( 16 \right) \end{aligned}$$

The equations for voltage-gated sodium channels were written as the following:

$$\begin{aligned} \alpha_{m}=q10\cdot0.32\cdot vtrap\alpha_{m}\left( V \right)\#\left( 17 \right) \end{aligned}$$

$$\begin{aligned} \beta_{m}=q10\cdot0.28\cdot vtrap\beta_{m}\left( V \right)\#\left( 18 \right) \end{aligned}$$

$$\begin{aligned} m_{\infty}=\frac{\alpha_{m}\left( V \right)}{\alpha_{m}\left( V \right)+ \beta_{m}\left( V \right)}\#\left( 19 \right) \end{aligned}$$

$$\begin{aligned} \tau_{m}=\frac{\frac{1}{\alpha_{m}\left( V \right)+ \beta_{m}\left( V \right)}}{1000}\#\left( 20 \right) \end{aligned}$$

The functions vtrapα_m_(V) and vtrapβ_m_(V) were written as follows:

vtrapα_m_(V) = function(voltage) vtrap((13.1-(voltage+65)), 4)

vtrapβ_m_(V) = function(voltage) vtrap(((voltage+65)-40.1), 5)

The function vtrap in these functions was written as follows:

vtrap <- function(x,y) {

if(x/y < 1E-6) {

result_vtrap <- y * (1-x/y/2)

} else {

result_vtrap <- x / (exp(x/y) - 1)

}

}

The gating parameter *h* was calculated using the following equations:

$$\begin{aligned} \alpha_{h}=q10\cdot0.128\cdot\exp\left( \frac{17-\left( V+65 \right)}{18} \right)\#\left( 21 \right) \end{aligned}$$

$$\begin{aligned} \beta_{h}=\frac{q10\cdot4}{\exp\left( \frac{40.0-\left( V+65 \right)}{5} \right)+1}\#\left( 22 \right) \end{aligned}$$

$$\begin{aligned} h_{\infty}=\frac{\alpha_{h}\left( V \right)}{\alpha_{h}\left( V \right)+ \beta_{h}\left( V \right)}\#\left( 23 \right) \end{aligned}$$

$$\begin{aligned} \tau_{h}=\frac{\frac{1}{\alpha_{h}\left( V \right)+ \beta_{h}\left( V \right)}}{1000}\#\left( 24 \right) \end{aligned}$$

The equations for high-threshold voltage-gated calcium channels were written as the following:

$$\begin{aligned} \alpha_{Ca\left( high \right)}=\frac{15.69\cdot\left( -V+81.5 \right)}{(\exp\left( \frac{-V+81.5}{10} \right)-1.0)}\#\left( 25 \right) \end{aligned}$$

$$\begin{aligned} \beta_{Ca\left( high \right)}=0.29\cdot\exp\left( -\frac{V}{10.86} \right)\#\left( 26 \right) \end{aligned}$$

$$\begin{aligned} c_{\infty}=\frac{\alpha_{Ca\left( high \right)}(V)}{\alpha_{Ca\left( high \right)}(V)+ \beta_{Ca\left( high \right)}(V)}\#\left( 27 \right) \end{aligned}$$

$$\begin{aligned} \tau_{Ca\left( high \right)}=\left( \frac{\frac{1}{tfa\cdot\left( \alpha_{Ca\left( high \right)}\left( V \right)+ \beta_{Ca\left( high \right)}\left( V \right) \right)}}{100} \right)\#\left( 28 \right) \end{aligned}$$

The equations for low-threshold voltage-gated calcium channels were written as the following:

$$\begin{aligned} \alpha_{Ca\left( low \right)}=\frac{0.055\cdot\left( -27.01-V \right)}{\left( \exp\left( \frac{-27.01-V}{3.8} \right)-1.0 \right)}\#\left( 29 \right) \end{aligned}$$

$$\begin{aligned} \beta_{Ca\left( low \right)}=0.94\cdot\exp\left( \frac{-63.01-V}{17} \right)\#\left( 30 \right) \end{aligned}$$

$$\begin{aligned} c2_{\infty}=\frac{\alpha_{Ca\left( low \right)}\left( V \right)}{\alpha_{Ca\left( low \right)}\left( V \right)+ \beta_{Ca\left( low \right)}\left( V \right)}\#\left( 31 \right) \end{aligned}$$

$$\begin{aligned} \tau_{Ca\left( low \right)}=\left( \frac{\frac{1}{tfa2\cdot\left( \alpha_{Ca\left( low \right)}\left( V \right)+ \beta_{Ca\left( low \right)}\left( V \right) \right)}}{100} \right)\#\left( 32 \right) \end{aligned}$$

The function ‘efun’ referenced in equations (6) and (7) in the Results section of the manuscript was written as follows:

efun <- function(z) {

if(z < 1E-4) {

efun <- 1 - z/2

} else {

efun <- z / (exp(z) - 1)

}

}
