# Supplementary material for: Control of Neuronal Excitability by Cell Surface Receptor Density and Phosphoinositide Metabolism
Source: Front Pharmacol. 2021 Apr 21;12:663840. doi: 10.3389/fphar.2021.663840 (PMC8097148; doi:10.3389/fphar.2021.663840)
Supplement: Supplementary file 2 [file table1.docx]

**Supplemental Table 1. Parameters for description of ion channel activities**

| **Parameter** | **Value** | **Reference** |
| --- | --- | --- |
| E_Na_ | 50 mV | (Traub et al., 1991; Zaika et al., 2006) |
| E_K_ | -80 mV | (Migliore et al., 1995; Zaika et al., 2006) |
| E_L_ | -50 mV | (Zaika et al., 2006) |
| g_Na_ | 4 mS/cm^2^ | (Traub et al., 1991; Zaika et al., 2006) |
| g_KDR_ | 0.8 mS/cm^2^ | (Migliore et al., 1995; Zaika et al., 2006) |
| g_KCNQ_ | 0.234 mS/cm^2^ | (Borg-Graham, 1991; Migliore et al., 1995) |
| g_KCNMA1_ | 8.8 mS/cm^2^ | (Moczydlowski and Latorre, 1983; Migliore et al., 1995) |
| g_Ca(low)_ | 0.6 mS/cm^2^ | (Somjen et al., 2008) |
| g_Ca(high)_ | 1 mS/cm^2^ | (Migliore et al., 1995; Zaika et al., 2006) |
| g_L_ | 0.4 mS/cm^2^ | (Zaika et al., 2006) |
| k_i_ | 0.001 | (Migliore et al., 1995) |
| f | 12.67056 | (Migliore et al., 1995) |
| K2f_ATPase_ | 0.00051137214 | (Quadroni and Knopfel, 1994) |
| f_ATPase_ | 100 | (Quadroni and Knopfel, 1994) |
| b_ATPase_ | 0.005 | (Quadroni and Knopfel, 1994) |
| K2f_ex_ | 6.3680304e-8 | (Quadroni and Knopfel, 1994) |
| E_1_ | 0.01315 | (Quadroni and Knopfel, 1994) |
| E_2_ | 0.0255 | (Quadroni and Knopfel, 1994) |
| Na_C_ | 7.6 mM | (Quadroni and Knopfel, 1994; Migliore et al., 1995) |
| Na_Ex_ | 152 mM | (Quadroni and Knopfel, 1994; Migliore et al., 1995) |
| Ca_Ex_ | 2.0 mM | (Quadroni and Knopfel, 1994; Migliore et al., 1995) |
| ζ_KM_ | -10 | (Borg-Graham, 1991; Migliore et al., 1995) |
| V_halfKM_ | -55 mV | (Borg-Graham, 1991; Migliore et al., 1995) |
| T | 297.15 K |  |
| gm_KM_ | 0.06 | (Borg-Graham, 1991; Migliore et al., 1995) |
| α0_KM_ | 0.006 | (Borg-Graham, 1991; Migliore et al., 1995) |
| q10_KM_ | 1.174619 | (Borg-Graham, 1991; Migliore et al., 1995) |
| abar | 0.28 | (Migliore et al., 1995) |
| k1 | 0.48e-3 | (Migliore et al., 1995) |
| d1 | 0.84 | (Migliore et al., 1995) |
| F | 96.520 C/mol |  |
| R | 8.313424 J/(K ⋅ mol) |  |
| bbar | 0.48 | (Migliore et al., 1995) |
| d2 | 1.0 | (Migliore et al., 1995) |
| k2 | 0.13e-6 | (Migliore et al., 1995) |
| ζn | -5.0 | (Migliore et al., 1995) |
| V_halfn_ | -32 mV | (Migliore et al., 1995) |
| gm_n_ | 0.4 | (Migliore et al., 1995) |
| q10 | 0.5172819 | (Migliore et al., 1995) |

| **Parameter** | **Value** | **Reference** |
| --- | --- | --- |
| α0_n_ | 0.03 | (Migliore et al., 1995) |
| V_halfl_ | -61 mV | (Migliore et al., 1995) |
| ζl | 2.0 | (Migliore et al., 1995) |
| gm_l_ | 1.0 | (Migliore et al., 1995) |
| α0_l_ | 0.001 | (Migliore et al., 1995) |
| tfa | 1.0 | (Migliore et al., 1995) |
| tfa2 | 5.0 | (Somjen et al., 2008) |

Borg-Graham, L. (1991). *Modelling the non-linear conductances of excitable membranes.* Oxford University Press.

Migliore, M., Cook, E.P., Jaffe, D.B., Turner, D.A., and Johnston, D. (1995). Computer simulations of morphologically reconstructed CA3 hippocampal neurons. *J Neurophysiol* 73(3)**,** 1157-1168.

Moczydlowski, E., and Latorre, R. (1983). Gating kinetics of Ca^2+^-activated K^+^ channels from rat muscle incorporated into planar lipid bilayers. Evidence for two voltage-dependent Ca^2+^ binding reactions. *J Gen Physiol* 82(4)**,** 511-542. doi: 10.1085/jgp.82.4.511.

Quadroni, R., and Knopfel, T. (1994). Compartmental models of type A and type B guinea pig medial vestibular neurons. *J Neurophysiol* 72(4)**,** 1911-1924. doi: 10.1152/jn.1994.72.4.1911.

Somjen, G.G., Kager, H., and Wadman, W.J. (2008). Computer simulations of neuron-glia interactions mediated by ion flux. *J Comput Neurosci* 25(2)**,** 349-365. doi: 10.1007/s10827-008-0083-9.

Traub, R.D., Wong, R.K., Miles, R., and Michelson, H. (1991). A model of a CA3 hippocampal pyramidal neuron incorporating voltage-clamp data on intrinsic conductances. *J Neurophysiol* 66(2)**,** 635-650. doi: 10.1152/jn.1991.66.2.635.

Zaika, O., Lara, L.S., Gamper, N., Hilgemann, D.W., Jaffe, D.B., and Shapiro, M.S. (2006). Angiotensin II regulates neuronal excitability via phosphatidylinositol 4,5-bisphosphate-dependent modulation of K_V_7 (M-type) K^+^ channels. *J Physiol* 575(Pt 1)**,** 49-67. doi: 10.1113/jphysiol.2006.114074.
