# Supplementary material for: Control of Neuronal Excitability by Cell Surface Receptor Density and Phosphoinositide Metabolism
Source: Front Pharmacol. 2021 Apr 21;12:663840. doi: 10.3389/fphar.2021.663840 (PMC8097148; doi:10.3389/fphar.2021.663840)
Supplement: Supplementary file 3 [file table2.docx]

**Supplemental Table 2. Rate constants and parameters for description of phosphoinositide metabolism of rat superior cervical ganglion neurons**

| **Parameter** | **Value** | **Reference** |
| --- | --- | --- |
| R (M_1_ density) | 15.87 μm^-2^ | (Kruse et al., 2016) |
| G (G protein density) | 40 μm^-2^ | (Falkenburger et al., 2013) |
| P (PLC density) | 3.12 μm^-2^ | (Kruse et al., 2016) |
| (free) PI(4,5)P_2_ (density) | 3,232 μm^-2^ | (Xu et al., 2003; Kruse et al., 2016) |
| Bound PI(4,5)P_2_ (density) | 6,464 μm^-2^ | (Kruse et al., 2016) |
| PI(4)P (density) | 4,540 μm^-2^ | (Xu et al., 2003; Kruse et al., 2016) |
| PI (density) | 226,975 μm^-2^ | (Xu et al., 2003; Kruse et al., 2016) |
| DAG (basal, plasma membrane) | 13 μm^-2^ | (Kruse et al., 2016) |
| Surface (area plasma membrane) | 4,100 μm^2^ | (Kruse et al., 2016) |
| size_cytosol (volume) | 6,644 μm^3^ | (Kruse et al., 2016) |
| size_ER (volume) | 1,196 μm^3^ | (Falkenburger et al., 2013; Kruse et al., 2016) |
| k_4K | 0.0008 s^-1^ | (Kruse et al., 2016) |
| k_4P | 0.12 s^-1^ | (Kruse et al., 2016) |
| k_5K | 0.02 s^-1^ | (Kruse et al., 2016) |
| k_5P | 0.028 s^-1^ | (Kruse et al., 2016) |
| k_PLC | 0.3 μm^2^ s^-1^ | (Kruse et al., 2016) |
| k_IP_3_ase | 0.13 s^-1^ | (Kruse et al., 2016) |
| k_DAGase | 0.20 s^-1^ | (Kruse et al., 2016) |
| Ca^2+^_buffer_high_affinity | 10 μΜ | (Kruse et al., 2016) |
| Ca^2+^_buffer_low_affinity | 20 μΜ | (Kruse et al., 2016) |
| Κ_D__Ca^2+^_high_affinity | 0.1 μΜ | (Wanaverbecq et al., 2003) |
| Κ_D__Ca^2+^_low_affinity | 1 μΜ | (Wanaverbecq et al., 2003) |

Falkenburger, B.H., Dickson, E.J., and Hille, B. (2013). Quantitative properties and receptor reserve of the DAG and PKC branch of G_q_-coupled receptor signaling. *The Journal of General Physiology* 141(5)**,** 537-555. doi: 10.1085/jgp.201210887.

Kruse, M., Vivas, O., Traynor-Kaplan, A., and Hille, B. (2016). Dynamics of Phosphoinositide-Dependent Signaling in Sympathetic Neurons. *J Neurosci* 36(4)**,** 1386-1400. doi: 10.1523/JNEUROSCI.3535-15.2016.

Wanaverbecq, N., Marsh, S.J., Al-Qatari, M., and Brown, D.A. (2003). The plasma membrane calcium-ATPase as a major mechanism for intracellular calcium regulation in neurones from the rat superior cervical ganglion. *J Physiol* 550(Pt 1)**,** 83-101. doi: 10.1113/jphysiol.2002.035782.

Xu, C., Watras, J., and Loew, L.M. (2003). Kinetic analysis of receptor-activated phosphoinositide turnover. *J Cell Biol* 161(4)**,** 779-791. doi: 10.1083/jcb.200301070.
